# AGAMOUS mediates timing of guard cell formation during gynoecium development

**DOI:** 10.1371/journal.pgen.1011000

**Published:** 2023-10-11

**Authors:** Ailbhe J. Brazel, Róisín Fattorini, Jesse McCarthy, Rainer Franzen, Florian Rümpler, George Coupland, Diarmuid S. Ó’Maoiléidigh

**Affiliations:** 1 Department of Biology, Maynooth University, Ireland; 2 The Max Plank Institute for Plant Breeding Research, Cologne, Germany; 3 Department of Biochemistry and Systems Biology, The University of Liverpool, United Kingdom; 4 Department of Genetics, Friedrich Schiller University Jena, Jena, Germany; National University of Singapore and Temasek Life Sciences Laboratory, SINGAPORE

## Abstract

In *Arabidopsis thaliana*, stomata are composed of two guard cells that control the aperture of a central pore to facilitate gas exchange between the plant and its environment, which is particularly important during photosynthesis. Although leaves are the primary photosynthetic organs of flowering plants, floral organs are also photosynthetically active. In the Brassicaceae, evidence suggests that silique photosynthesis is important for optimal seed oil content. A group of transcription factors containing MADS DNA binding domains is necessary and sufficient to confer floral organ identity. Elegant models, such as the ABCE model of flower development and the floral quartet model, have been instrumental in describing the molecular mechanisms by which these floral organ identity proteins govern flower development. However, we lack a complete understanding of how the floral organ identity genes interact with the underlying leaf development program. Here, we show that the MADS domain transcription factor AGAMOUS (AG) represses stomatal development on the gynoecial valves, so that maturation of stomatal complexes coincides with fertilization. We present evidence that this regulation by AG is mediated by direct transcriptional repression of a master regulator of the stomatal lineage, *MUTE*, and show data that suggests this interaction is conserved among several members of the Brassicaceae. This work extends our understanding of the mechanisms underlying floral organ formation and provides a framework to decipher the mechanisms that control floral organ photosynthesis.

## Author summary

Photosynthesis supports the growth and development of plants and is mainly associated with leaves. However, other organs, including floral organs, are photosynthetic and contribute to the energy requirements of the plant, which has attracted the interest of crop breeders. Despite its importance, very little is known about the establishment and molecular regulation of floral organ photosynthesis. Here, we describe the developmental progression and regulation of stomatal formation on the female reproductive organs of *Arabidopsis thaliana* before and after fertilization. Stomata control gas exchange between the plant and the environment, which facilitates photosynthesis and transpiration. We show that stomatal formation coincides with fertilization of the flower but does not depend upon it. Instead, the activity of a master regulator of stomatal development is directly controlled by a flower-specific factor. We present evidence that this regulation is conserved in several members of the mustard plant family (Brassicaceae) and suggest that the timing of stomatal formation on the female reproductive organs may be modified between species, which may have adaptive benefits. Given that floral organs are derived from leaves, this work also broadens and deepens our understanding of how the underlying leaf developmental program is rewired during flower development.

## Introduction

In eudicots, such as *Arabidopsis thaliana*, flowers are composed of four types of floral organs: sepals, petals, stamens, and carpels. The sepals contain high levels of chlorophyll and bear stomata, making them leaf-like in appearance. In contrast, mature petals and stamens lack substantial concentrations of chlorophyll [1,2]. Stomata are absent from petals but modified stomata are present on the abaxial surfaces of anthers [1,3]. In *A*. *thaliana*, the ovules are encased within the gynoecium which is formed of two fused carpels and other tissues that arise from the carpels, such as the style, stigma and replum [1,4] (S1A Fig). Although the gynoecial valves lack stomata, they are present on the differentiated and elongated silique valve epidermis [5,6]. Stomata on siliques enable atmospheric carbon fixation to support photosynthesis, and photosynthetic activity of siliques has been demonstrated in several members of the Brassicaceae family including *A*. *thaliana* [7–10]. This photosynthetic activity positively influences seed oil content and is of interest to crop breeders [8,9,11]. Stomata also support transpiration, which drives the movement of nutrients through the plant and simultaneously facilitates cooling [12]. However, very little is known about the molecular mechanisms of stomatal development on these organs.

On leaves of *A*. *thaliana*, the stomatal cell lineage is initiated by the asymmetric cell division of a protodermal cell (or meristemoid mother cell), which is regulated by the basic helix-loop-helix (bHLH) transcription factor SPEECHLESS (SPCH) [13–15] (S1B Fig). This asymmetric division produces a meristemoid and a stomatal lineage ground cell (SLGC). The meristemoid may then undergo further asymmetric cell divisions to renew itself and produce more SLGCs. Alternatively, a meristemoid may transition into a rounded cell termed a guard mother cell (GMC) [14,15]. The transition from meristemoid to GMC is coordinated by another bHLH transcription factor, MUTE, which also functions to attenuate asymmetric cell divisions [16]. GMCs then divide symmetrically to produce two guards cells and ultimately a mature stomatal complex, a process that is mediated by a third bHLH transcription factor, FAMA [17]. Two other bHLH transcription factors, SCREAM (SCRM) and SCRM2, form heterodimers with SPCH, MUTE, and FAMA to coordinate gene expression [18]. These division steps are regulated such that stomatal complexes are separated by at least one cell, which ensures control of the central pore aperture [14,15]. Several transmembrane receptors have been implicated in this stomatal patterning, such as TOO MANY MOUTHS (TMM), ERECTA (ER), ER-LIKE1 (ERL1), and ERL2 [15,19,20]. The activities of these receptors are modulated by the EPIDERMAL PATTERNING FACTORs (EPFs) and EPF-LIKES (EPFLs) secreted peptide families (S1B Fig) [15,21–23].

Floral organ identity is controlled by a group of transcription factors that contain MADS DNA-binding domains [24–28]. In the absence of their activities, floral organs are converted into leaf-like organs while ectopic expression of these transcription factors is sufficient to transform leaves into floral organs [28–32]. These observations confirmed a long-standing hypothesis that floral organs are derived from leaves [33]. They also formed the basis of the ABCE model of flower development and the floral quartet model, which largely address organ specification [28–32,34]. However, these MADS domain transcription factors continue to be expressed after the floral organs have been specified and they are known to control the expression of genes required for differentiation [35–38]. Identifying the repertoire of differentiation processes that the floral organ identity genes control remains a key challenge [25].

The MADS-domain protein AGAMOUS (AG) controls the specification of stamens and carpels and is required for floral meristem termination [27]. AG interacts with E-class proteins, SEPALLATA 1–4 (SEP1-4), in heterodimeric or tetrameric complexes to coordinate carpel specification and floral meristem termination, with SEP3 playing an especially important role [29,34,39]. *AG* activity promotes carpel development in a partially redundant manner with its closest related paralogs *SHATTERPROOF1* (*SHP1*) and *SHP2* [40]. AG and the SHP proteins also suppress the formation of epidermal hairs (trichomes) during carpel differentiation, which represents the first tangible example of how the floral organ identity proteins modify the underlying leaf development program to generate floral organs [35,41].

Here, we investigated the developmental progression and molecular regulation of stomatal development on gynoecial and silique valves in *A*. *thaliana*. We describe the normal progression of stomatal development before and after fertilization, and present evidence that AG suppresses this process in *A*. *thaliana* and other members of the Brassicaceae. The data presented provide further evidence and mechanism that transcription factors conferring floral organ identity directly suppress aspects of leaf development during floral organ formation. They also provide a framework with which to understand the establishment of the silique as a photosynthetic organ in the Brassicaceae.

## Results

### Stomatal development on gynoecial valves

As mentioned above, the photosynthetic activity of siliques of the Brassicaceae family contributes significantly to the carbon requirements of seeds [8,9,11]. Siliques assimilate atmospheric CO_2_ [7,10,11], probably through the stomata present on the valve epidermis, which are not present on pre-anthesis gynoecial valves (S1C Fig) [1]. The presence of stomata on floral organs was previously reported [1,3,5] (S1D–S1F Fig), however, the progression of stomatal development on these organs has not been fully described. We addressed this knowledge gap by examining the developmental progression of stomata on the siliques of *A*. *thaliana*.

**Fig 1:**
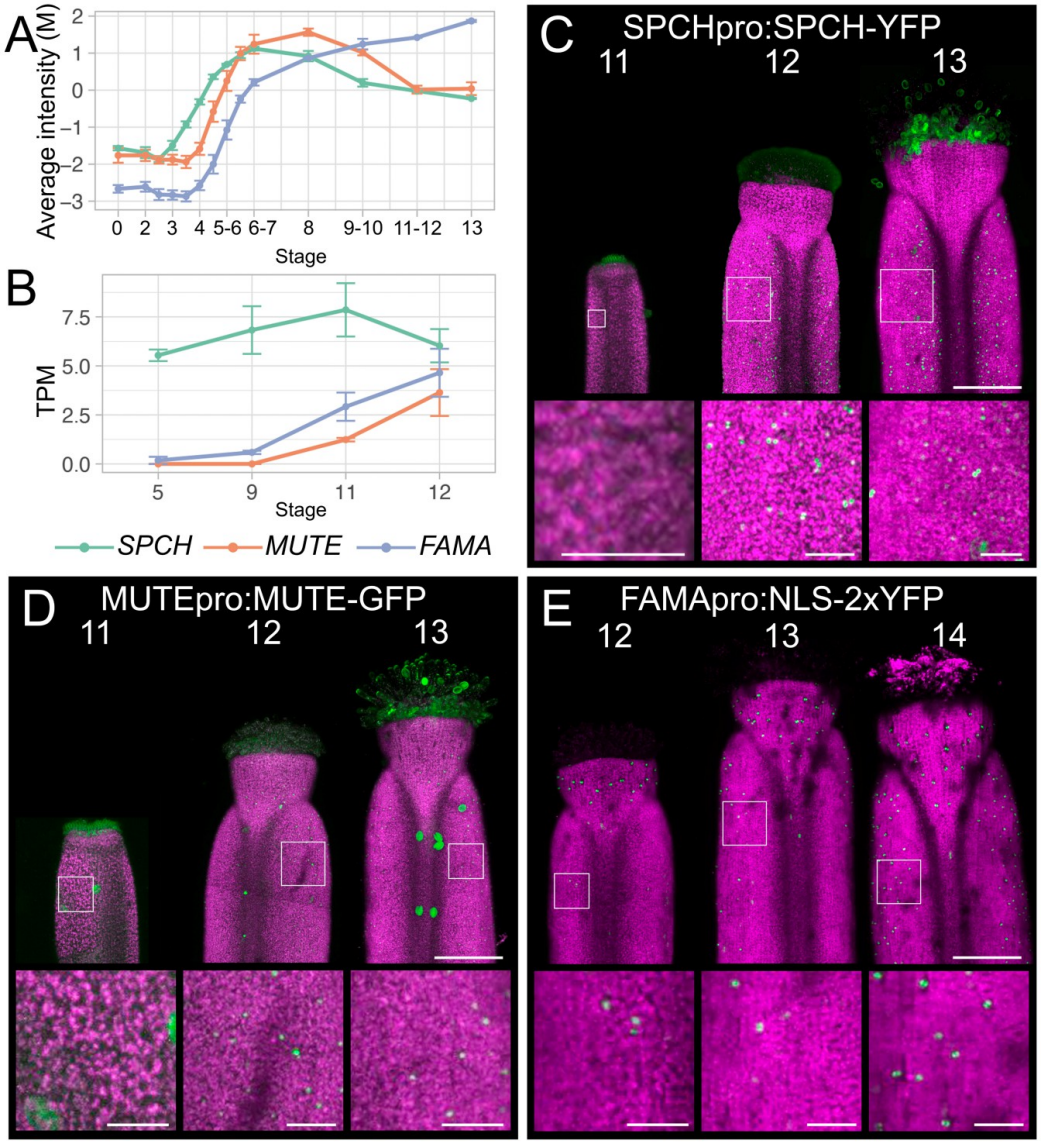
Expression of stomatal bHLH transcription factors during gynoecium development. (A-B) Levels of *SPCH*, *MUTE*, and *FAMA* mRNAs at described stages of flower development in (A) whole flower buds [42] and (B) laser-microdissected gynoecia [43]. Error bars in (A-B) are s.e.m of three and four independent biological replicates, respectively. M, log-ratio of time-point to a common reference; TPM, transcripts per million. (C-E) Maximum intensity projections of stitched confocal laser scanning z-stacked micrographs of (C) *SPCHpro*:*SPCH-YFP*, (D) *MUTEpro*:*MUTE-GFP*, (E) *FAMApro*:*NLS-2xYFP* transgenes at different stages of gynoecium development as indicated. White boxes indicate areas that were magnified to produce the insets. Fluorescent protein is colored green and chlorophyll fluorescence is colored magenta. Scale bars for images of whole gynoecia are 100 μm. Scale bars for insets are 20 μm.

We started by surveying the mRNA levels of master regulators of stomatal development as reported by publicly available transcriptomics datasets of flower and gynoecium development (Fig 1A and 1B). We focused on the expression of three bHLH transcription factor-coding genes, *SPCH*, *MUTE*, and *FAMA*, which are necessary and sufficient to promote different stages of the stomatal lineage [13,16,17]. A distinct peak of *SPCH* and *MUTE* mRNA was detected at stage 6–7 and 8 of flower development, respectively, in genome-wide expression profiling using a synchronous flowering system (Fig 1A) [42]. These peaks corresponded to the stages of initiation and progression of the stomatal lineage on sepals, which began to mature at approximately stage 10 (S1D Fig). Levels of both *SPCH* and *MUTE* mRNA then decreased until anthesis, although *MUTE* levels plateaued between stages 11 and 13. In contrast, *FAMA* levels continued to increase as flower development progressed (Fig 1A). More recent transcriptomics datasets derived from laser-microdissected gynoecia at different stages of flower development provided improved spatial resolution [43]. These data are not complicated by the presence of other floral organs that bear stomata, such as the sepals and stamens, although the latest stage analyzed was stage 12 of flower development. *SPCH* mRNA levels were relatively high from approximately stage 5 and rose slightly until stage 11 and then decreased (Fig 1B). *MUTE* mRNA was not detectable before stage 11 when it was weakly expressed and then increased ~3-fold by stage 12 (Fig 1B). *FAMA* mRNA was also not detected at stage 5 but increased steadily by ~8-fold between stages 9 and 12 (Fig 1B). Based on these data, we concluded that *MUTE* and *FAMA* transcription in the gynoecium was initiated at approximately stage 11–12, whereas *SPCH* transcription was initiated earlier.

To cross-examine these transcriptomic data and to provide a better resolution of the expression of these genes in the gynoecium, we obtained transgenic lines that harbor fluorescent translational or transcriptional reporters for *SPCH* [44], *MUTE* [16], and *FAMA* [45] whose transcription is driven by their endogenous regulatory elements (Fig 1C–1E). Using laser-scanning confocal microscopy, we could not detect SPCH-YFP in stage 11 gynoecial valves, but the SPCH-YFP protein was abundant in the epidermis of late stage 12 and stage 13 gynoecial valves (Fig 1C). The contrast between mRNA and protein accumulation may indicate the presence of a post-translational mechanism to control SPCH protein levels (Fig 1B and 1C). A pattern of protein accumulation was found for MUTE-GFP, which was largely in agreement with the transcriptomics data in gynoecia (Fig 1B and 1D). The accumulation of YFP from the *FAMApro*:*NLS-2xYFP* transgene was delayed relative to either of these reporters, with few fluorescent foci present at stage 12, which became slightly more abundant at stage 13 (Fig 1E). At stage 14, accumulation of YFP was observed as fluorescent foci throughout the gynoecial valve (Fig 1E). Therefore, the SPCH and MUTE proteins accumulate after stage 11 but before late stage 12, whereas *FAMA* transcription is initiated in the valves from late stage 12.

**Fig 2:**
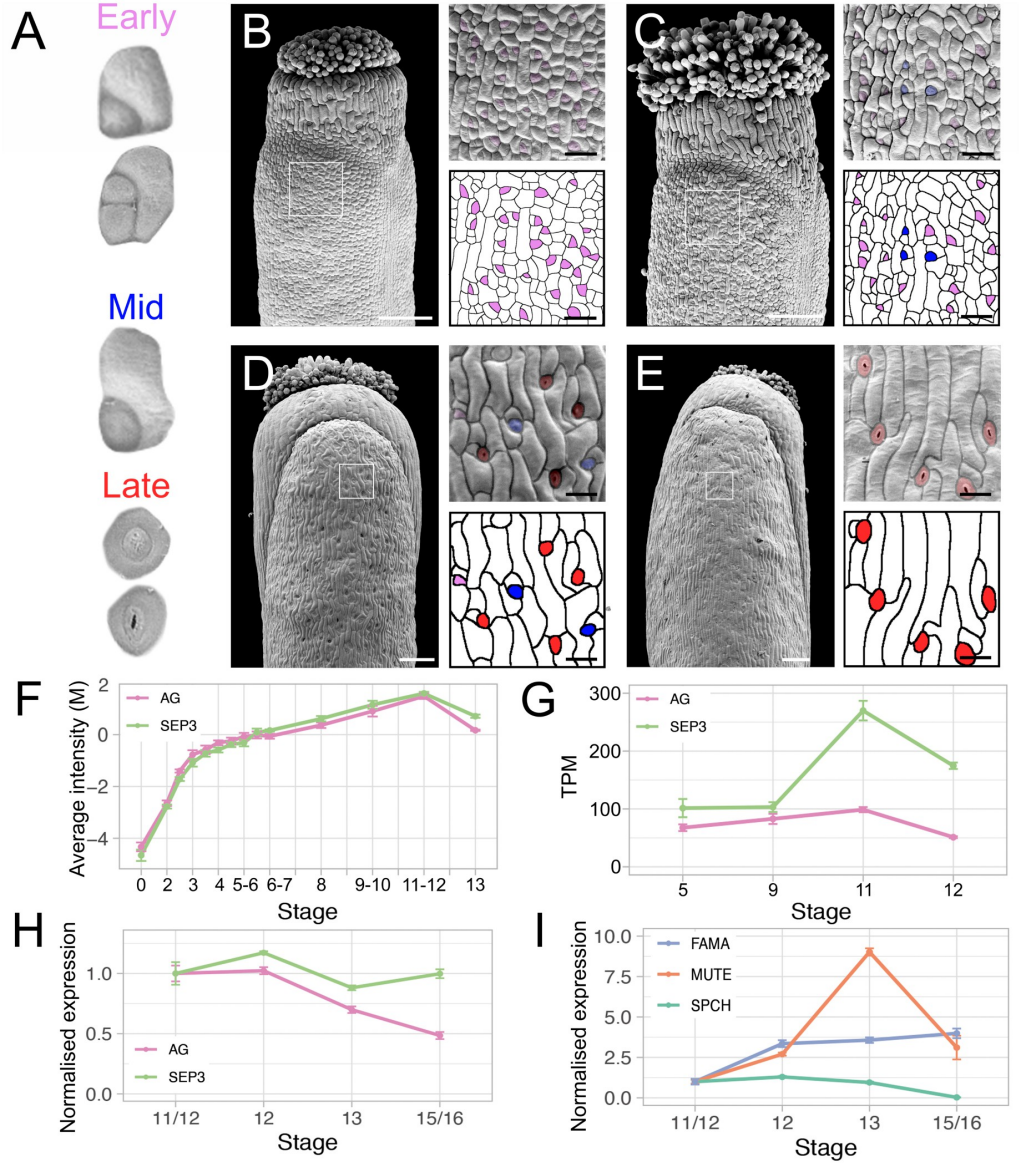
Progression of stomatal development on gynoecial and silique valves. (A) Example stomatal lineage cells used to define early, mid, and late stages of stomatal development for morphological analysis (not to scale). Cells of the stomatal lineage are distinguished based on the presence of an asymmetric cell division and/or presumed early stage GMCs (Early stage), the rounding of a cell similar to a GMC (Mid stage), and the presence of a symmetric cleavage (Late stage). (B-E) Scanning electron micrographs of gynoecia at floral stage (B) 12, (C) 13, (D) 15–16 gynoecia and (E) a stage >17 silique with magnifications of the cell surface (from white box). Scale bars for images of whole gynoecia are 100 μm. Scale bars for magnifications are 20 μm. Purple, blue, and red highlights indicate early, mid, and late stage stomatal lineage morphology, respectively. (F-H) Levels of *AG* and *SEP3* mRNAs over the course of (F) flower development determined by microarray analysis [42] and (G) gynoecium development determined by laser-capture microdissection combined with RNA-Seq [43] and (G) gynoecium development determined by RT-qPCR. (I) Levels of *SPCH*, *MUTE*, and *FAMA* mRNAs during gynoecium development determined by RT-qPCR. Error bars in (F-I) are s.e.m of (F-G) three and (H-I) four independent biological replicates, respectively. M, log-ratio of time-point to a reference; TPM, transcripts per million. Expression in (H-I) was normalized to the average of late stage 11/early stage 12 gynoecia time-point.

We then examined the formation of stomatal cell lineage types on gynoecial valves using scanning electron microscopy (SEM). Early asymmetric cell divisions (i.e., meristemoids) were readily identified, however, it somewhat ambiguous when these meristemoids started to transition to GMCs. Therefore, we classified meristemoid and presumed early GMCs as ‘early’ stomatal lineage cells (Fig 2A). The presence of conspicuous symmetric cell divisions allowed us to unambiguously identify late-stage GMCs (Fig 2A). Similarly, the presence of open stomatal pores clearly identified mature stomates (Fig 2A). We classified both late-stage GMCs and mature stomates as ‘late’ stomatal lineage cells (Fig 2A). ‘Mid’ stage cells GMCs were in turn defined by the rounding of the stomatal lineage cell and the absence of a symmetric cell division (Fig 2A). To validate this morphological analysis, we performed an unbiased assessment of the stomatal lineage through cell size analysis, which can be interpreted to also separate the stomatal lineage cells into early (<25 μm^2^), mid (25–40 μm^2^), and late (>40 μm^2^) stages (see Materials and methods for details). These two methods produced very similar trends of stomatal progression through the stages of gynoecium and silique development (S2A and S2B Fig, S1 and S2 Tables).

We observed early-stage stomatal lineage cells on gynoecial valves during stage 12 of flower development whereas late-stage stomatal cell lineages were largely absent and mid-stage cells comprised a small fraction of the total stomatal cell lineage cells (Fig 2B, S2A and S2B Fig, S1 and S2 Tables). At stage 13, early-stage lineage cells increased in their abundance and late-stage cells began to appear whereas mid-stage cells remained a small fraction of the total stomatal cell linage cells (Fig 2C, S2A and S2B Fig, S1 and S2 Tables). Post-fertilization, at stage 15–16, the frequency of early-stage cells reduced dramatically in comparison to stage 13 with a commensurate increase in mid-stage and late-stage cells (Fig 2D, S2A and S2B Fig, S1 and S2 Tables), until most stomatal lineage cells were of late-stage on older (>stage 17) siliques (Fig 2E, S2A and S2B Fig, S1 and S2 Tables).

Stomata also formed on the style of the gynoecium prior to the formation of stomata on the valves, with mature stomata present on the style of stage 12 gynoecia (S1C Fig). However, the style comprises only a small fraction of the total area of the mature silique (S1F Fig), suggesting that stomata on the style contribute only a small fraction of the total carbon fixation and transpiration capacity of the silique. Given the close association between fertilization and progression of stomatal development, we tested whether a dependent relationship existed (S2C and S2D Fig). To this end, we emasculated flowers before anthesis and examined gynoecial valves 5 d after anthesis by SEM (S2C Fig). We found mature stomatal complexes on these unfertilized gynoecial valves (S2D Fig), which indicated that maturation of stomatal complexes on the gynoecium does not depend on fertilization.

### Expression of *AG* and *SEP3* during the formation of stomatal complexes

The C class protein AG controls the specification and development of stamens and carpels by forming dimers or tetramers with the E class protein SEP3 [28–30,46]. Notably, it has been shown that AG suppresses the formation of trichomes on carpel valves by controlling the expression of other developmental regulators [35,41]. In addition, trichomes form on the gynoecia of plants with reduced *SEP* activity [32]. Because stomata, like trichomes, are formed by the epidermis, we hypothesized that an AG-SEP3 complex might also repress the formation of stomata on carpel valves. To explore this, we first assessed published expression data for *AG* and *SEP3* in gynoecial tissues over the course of stomatal initiation and maturation. *In situ* hybridizations showed *AG* mRNA accumulation throughout the carpels during stage 8 before its expression becomes restricted and absent from stage 12 gynoecial valves [47]. A similar expression pattern was observed for *SEP3* mRNA in the gynoecium, although its expression was restricted from gynoecial valves from approximately stage 10 [48]. This is consistent with the results of transcriptomics experiments, where *AG* and *SEP3* mRNA levels begin to decrease from approximately stage 11 of flower development (Fig 2F and 2G) [42,43].

AG-GFP and SEP3-GFP protein accumulation was analyzed in transgenic plants that harbored *AGpro*:*AG-GFP* and *SEP3pro*:*SEP3-GFP* transgenes that drive transcription from the corresponding endogenous regulatory elements [49]. AG-GFP fluorescence was observed throughout stage 12 gynoecial valves in these plants even though *AG* mRNA is expressed at a low levels in the valves at this stage [47], possibly due to the low turnover of AG at these stages [49]. We verified these results for AG-GFP using a similar but independently generated *AGpro*:*AG-GFP* transgenic line using laser-scanning confocal microscopy (S3A and S2B Fig) [41]. We also imaged stage 13 gynoecia and found that fluorescent signal stemming from AG-GFP was more intense in the replum and style relative to the valves (S3C Fig). Additionally, SEP3-GFP protein accumulated to much higher levels in the replum and valve margins at stage 12 when compared to the gynoecial valves [49]. Taken together with the transcriptomics data, it appears that the abundance of AG and SEP3 proteins in the gynoecial valves reduces significantly between stages 11 and 13 of flower development.

To further assess the mRNA levels of *AG*, *SEP3*, *SPCH*, *MUTE*, and *FAMA*, we harvested gynoecia from late stage 11/early stage 12, late stage 12, stage 13 and stage 15–16 wild-type L-*er* plants and performed reverse-transcription combined with quantitative PCR (RT-qPCR) on the processed material (Fig 2H and 2I, S3 Table). In our experiment, expression of *AG* and *SEP3* was largely similar between late stage 11/early stage 12 and late stage 12 (*p*.*adj* = 0.628 and *p*.*adj* = 0.25, respectively, pairwise paired two-tailed t-tests). *AG* mRNA and *SEP3* levels then decreased between late stage 12 and 13 to 0.67 and 0.75-fold, respectively (*p*.*adj* = 0.006 and *p*.*adj* = 0.005, respectively, pairwise paired two-tailed t-tests) (Fig 2H, S3 Table). *AG* levels decreased further between stage 13 and 15–16 by 0.69-fold (*p*.*adj* = 0.004, pairwise paired two-tailed t-test) whereas mean *SEP3* levels increased slightly (*p*.*adj* = 0.107, pairwise paired two-tailed t-test), which is probably due to *SEP3* expression in the ovules/developing seeds (Fig 2H, S3 Table) [48]. *SPCH* mRNA levels increased slightly between late stage 11/early stage 12 and late stage 12 (*p*.*adj* = 0.023, pairwise paired two-tailed t-test) and then gradually fell between late stage 12 and stage 13 (*p*.*adj* = 0.007, pairwise paired two-tailed t-test) and between stage 13 and 15 (*p*.*adj* = 0.001, pairwise paired two-tailed t-test) (Fig 2I). Between late stage 11/early stage 12 and late stage 12 *MUTE* levels increased by 2.7-fold, mimicking its expression in the laser-capture microdissection data (*p*.*adj* = 0.001, pairwise paired two-tailed t-test) (Figs 1B and 2I). *MUTE* levels then increased by approximately 3.3-fold between late stage 12 and 13 (*p*.*adj* = 0.001, pairwise paired two-tailed t-test) and fell again by a similar magnitude between stage 13 and 15–16 (*p*.*adj* = 0.011, pairwise paired two-tailed t-test) (Fig 2I). Mean *FAMA* levels increased by 3.36-fold between late stage 11/early stage 12 and late stage 12 (*p*.*adj* = 0.002, pairwise paired two-tailed t-test) and steadily increased at stage 13 (3.57-fold, *p*.*adj* = 0.009, pairwise paired two-tailed t-test) and stage 15–16 (3.99-fold, *p*.*adj* = 0.015, pairwise paired two-tailed t-test) relative to late stage 11/early stage 12 (Fig 2I). Although the initial increase in *FAMA* expression between late stage 11/early stage 12 and late stage 12 was similar to the observations of the laser-capture microdissection data (Fig 1B), the subsequent increases were more gradual than expected given the prominent peak in *MUTE* expression observed at stage 13 (Fig 2I). Together, these data supported an anti-correlation between the *AG* and *SEP3* mRNA levels and *MUTE* expression between late stage 12 and stage 13 of flower development.

### Transcriptional response of master regulators of stomatal development to repression of *AG* activity

**Fig 3:**
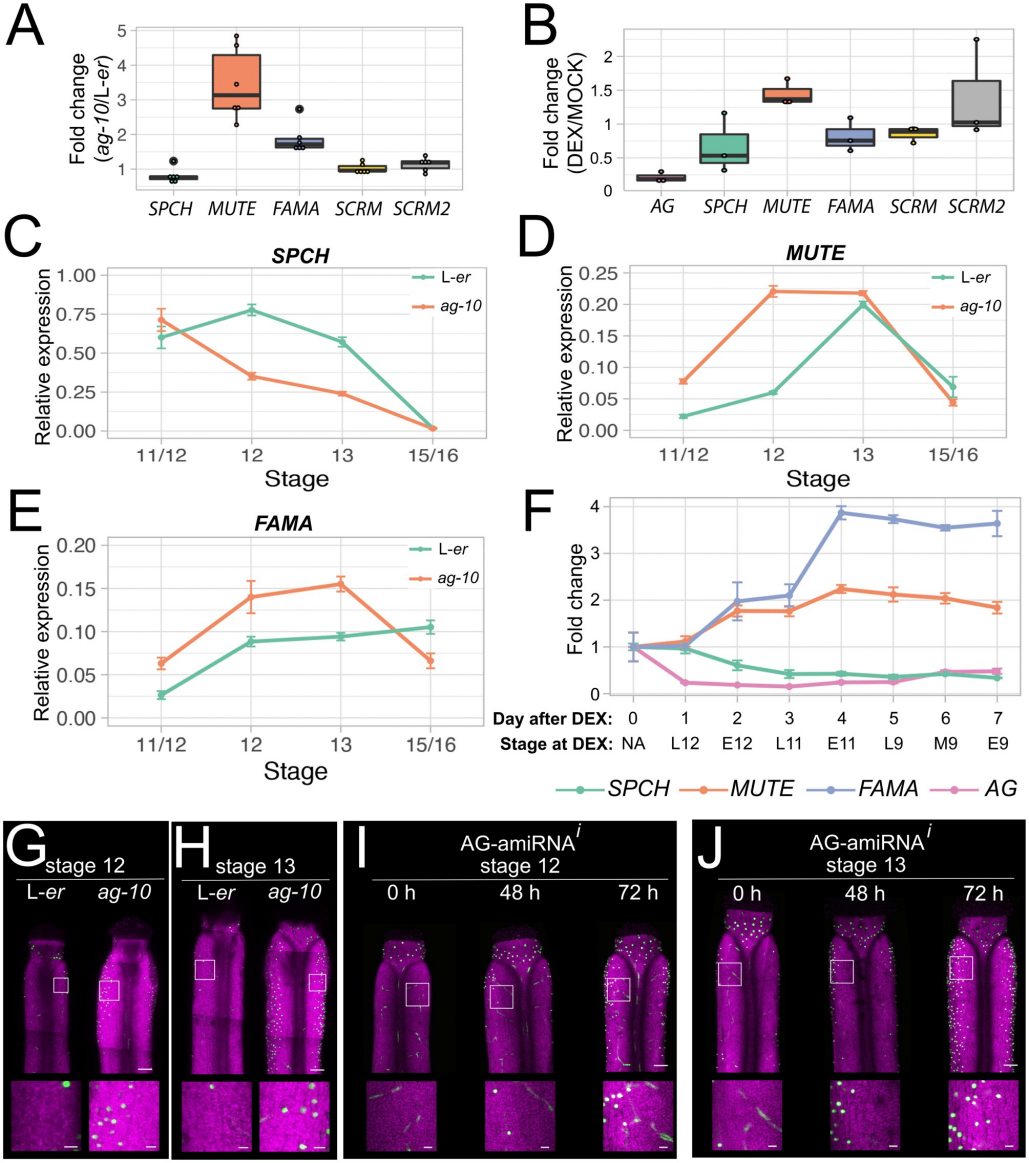
Transcriptional response of master regulators of stomatal development to repression of *AG* activity. (A-B) Levels of mRNAs encoding stomatal bHLH transcription factor regulators, as determined by RT-qPCR, in (A) *ag-10* stage 10–13 gynoecia relative to L-*er* stage 10–13 gynoecia, (B) dexamethasone-treated *AG-amiRNA*^*i*^ (*OPpro*:*AG-amiRNA/35Spro*:*GR-LhG4*) in stage 10–13 gynoecia relative to mock-treated *AG-amiRNA*^*i*^ in stage 10–13 gynoecia 24 h after treatments. Each dot in (A-B) represents the technical mean of an individual independent biological replicate. (C-E) Levels of (C) *SPCH*, (D) *MUTE*, and (E) *FAMA* mRNAs during gynoecium development as determined by RT-qPCR. Data for L-*er* is the same as in Fig 2I but was originally paired with the *ag-10* experiments presented in this figure. Errors bars are s.e.m. of four independent biological replicates (F) Levels of *SPCH*, *MUTE*, *FAMA*, and *AG* mRNAs, as determined by RT-qPCR, in stage 13 gynoecia after treatment with dexamethasone relative to untreated (0 d) in stage 13 gynoecia. “Day after DEX” indicates the number of days that gynoecia were treated with DEX before being harvested at anthesis (stage 13), with “0 d” representing the untreated sample. “Stage at DEX” indicates the approximate stage of the flower/gynoecium when DEX treatment was applied. Error bars are s.e.m of three independent biological replicates. (G-J) Maximum intensity projections of stitched confocal laser scanning z-stack micrographs of (G, I) stage 12 and (H, J) stage 13 gynoecia from plants harboring a *FAMApro*:*2xYFP* transgene in (G-H) L-*er* and *ag-10* backgrounds, and (I-J) the *AG-amiRNA*^*i*^ (*OPpro*:*AG-amiRNA/35Spro*:*GR-LhG4*) background before treatment (0 h) and after dexamethasone treatment (48 h and 72 h). YFP is colored green and chlorophyll fluorescence is colored magenta. Scale bars for images of whole gynoecia are 100 μm. Scale bars for insets are 20 μm.

To test whether a reduction of *AG* activity would result in the differential expression of master regulators of stomatal development, we harvested stage ~10–13 gynoecia from L-*er* wild-type plants harboring a fully functional copy of *AG* and those that were homozygous for the weak *ag-10* mutation [50]. Plants that are homozygous for *ag-10* form gynoecia that are relatively normal in appearance, although they tend to bulge [50]. We extracted total RNA from the gynoecia of these plants and using RT-qPCR, we found that the mean mRNA levels of *MUTE* and *FAMA* were increased in *ag-10* carpels to ~3.2 (*p* = 0.0002, two-tailed paired t-test) and ~1.8-fold (*p* = 0.0004, two-tailed paired t-test), respectively, relative to wild-type counterparts (Fig 3A, S3 Table). In contrast, the mean levels of *SPCH* mRNA were mildly decreased to 0.8-fold (*p* = 0.09, two-tailed paired t-test) while the mean mRNA levels of *SCRM* and *SCRM2* appeared unchanged (Fig 3A, S3 Table). Next, we used a transgenic line that harbors a dexamethasone (DEX) inducible artificial microRNA that was designed to target the *AG* mRNA (*AG-amiRNA*^*i*^) [41]. We treated the inflorescences of these plants with a DEX-containing solution and a mock solution, and harvested stage ~10–13 gynoecia 24 h later. By RT-qPCR, we found that mean *MUTE* mRNA levels were increased to ~1.4-fold (*p* = 0.08, two-tailed paired t-test) in DEX-treated samples relative to the mock control (Fig 3B, S3 Table), consistent with the increased expression detected in *ag-10*. In contrast, *SPCH*, *FAMA*, *SCRM*, and *SCRM2* were not consistently differentially expressed (Fig 3B, S3 Table).

We then examined the mRNA levels of *SPCH*, *MUTE*, and *FAMA* in late stage 11/early stage 12, late stage 12, stage 13, and stage 15–16 gynoecia/siliques derived from *ag-10* plants, which were harvested in a paired fashion with the L-*er* samples described above (Fig 2H and 2I). We found that *SPCH* levels were decreased to ~0.45 and ~0.42 fold in *ag-10* in comparison to L-*er* at late stage 12 and 13 (*p* = 0.0001 and 0.002, respectively, paired two-tailed t-tests) (Fig 3C, S3 Table). In contrast, *MUTE* levels were increased ~3.5-fold in late stage 11/early stage 12 (*p* = 0.002, paired two-tailed t-test) and ~3.7-fold in late stage 12 samples (*p* = 0.001, paired two-tailed t-test) (Fig 3D, S3 Table). Similarly, *FAMA* levels were increased ~2.4-fold in late stage 11/early stage 12 (*p* = 0.003, paired two-tailed t-test) and ~1.6-fold in late stage 12 samples (*p* = 0.03, paired two-tailed t-test) (Fig 3E, S3 Table). We then used the *AG-amiRNA*^*i*^ line in a time-course experiment where we harvested stage 13 gynoecia of untreated plants (0 d) and then treated the inflorescences of plants with a DEX-containing solution. We harvested only gynoecia that had reached anthesis every day for seven days following that treatment. Therefore, gynoecia that were harvested 1 d following DEX-treatment would have been ~late-stage 12 gynoecia at the time of DEX treatment. Gynoecia harvested at 2 d following DEX-treatment would have been ~early stage 12 gynoecia, and so on (Fig 3F, S3 Table). Following harvesting, we processed the samples and determined mRNA levels of *SPCH*, *MUTE*, *FAMA*, and *AG* using RT-qPCR. One day after treatment, there was very little change in the levels of *SPCH*, *FAMA*, or *MUTE* mRNAs whereas *AG* mRNA was reduced to 0.2-fold when compared with 0 d (*p*.*adj* < 10^−9^, pairwise two-tailed t-test), as has been previously described (Fig 3F, S3 Table) [41]. The lack of response from the bHLH-encoding genes may have been because the *AG-amiRNA* was activated in late-stage 12 gynoecia when *AG* mRNA levels were already very reduced in the gynoecial valve, although *AG* mRNA continued to accumulate in other tissues [47]. This contrasts with the L-*er/ag-10* RT-qPCRs where we observed accumulating defects derived from the static *ag-10* mutation (Fig 3C–3E). At 2 d post-induction, which roughly corresponded to activation of the *AG-amiRNA* in early stage 12 gynoecia, the mRNA levels of both *MUTE* and *FAMA* increased to ~1.8 (*p*.*adj* = 0.006, pairwise two-tailed t-test) and ~2-fold (*p*.*adj* = 0.02, pairwise two-tailed t-test) relative to 0 d, respectively, as *SPCH* mRNA levels decreased to ~0.6-fold (*p*.*adj* = 0.006, pairwise two-tailed t-test) (Fig 3F, S3 Table). *MUTE* and *FAMA* expression then continued to increase until 4 d post-induction where they peaked at ~2.2 (*p*.*adj* = 0.0004, pairwise two-tailed t-test) and ~3.9-fold (*p*.*adj* < 10^−5^, pairwise two-tailed t-test) relative to 0 d, respectively, and then began to decrease. At 5 d post-induction, *AG* mRNA levels began to recover, which correlated with a reduction in *MUTE* mRNA levels, which *FAMA* partially mimics, until the end of the experiment at 7 d post induction (Fig 3F, S3 Table). Taken together, we concluded that AG represses the expression of *MUTE*, and *FAMA* during gynoecium development.

To improve our understanding of the spatial resolution with which AG controls the expression of these stomatal genes, we introduced the *FAMApro*:*2xYFP* reporter into the *ag-10* background and imaged gynoecia at late stage 12 and stage 13 using confocal laser scanning microscopy [51]. We observed very few fluorescent foci on the valves of stage 12 wild-type gynoecia and stage 13 valves (Fig 3G and 3H, S4 Table), as previously described for the *FAMApro*:*NLS-2xYFP* reporter (Fig 1E). In contrast, ~23 times the number of foci were observed on the valves of stage 12 (*p* = 0.08, two-tailed paired t-test) and ~26 times the number of foci on stage 13 *ag-10* gynoecia (*p* = 0.01, two-tailed paired t-test) (Fig 3G and 3H, S4 Table). We then introduced the *FAMApro*:*2xYFP* reporter into the *AG-amiRNA*^*i*^ transgenic line. We treated inflorescences with a DEX-containing or mock solution and imaged stage 12 and 13 gynoecia every day for 3 days (Fig 3I and 3J and S4 Fig, S4 Table). Like the *ag-10* observations (Fig 3G and 3H) and mirroring the results of the RT-qPCR experiments (Fig 3F), fluorescent foci were prevalent on valves of the DEX-treated gynoecia at 48 and 72 h (Fig 3I and 3J). Far fewer fluorescent foci were observed on the valves of untreated samples (0 h, Fig 3I and 3J), mock-treated counterparts (S4B and S4D Fig), or DEX-treated samples after 24 h (S4A and S4C Fig). Each comparison within stages and the 48 h and 72 h time-points revealed unambiguous differences between DEX and mock-treated plants (*p* = 0.01–0.048, unpaired two-tailed t-tests) (S4 Table). When all stages and the 48 h and 72 h time-points were combined, DEX-treated gynoecia bore ~9 times the number of fluorescent foci relative to mock-treated counterparts (*p* = 0.01, unpaired two-tailed t-test) (S4 Table). These confocal data support the observations of the RT-qPCR experiments and clarify that expression of *FAMA* is not elevated throughout the gynoecial epidermis but only in a pattern that is consistent with stomatal formation on the gynoecial valves.

We initially focused our analysis on the *FAMApro*:*2xYFP* reporter as it was easily visualized, and we reasoned that an increase in *MUTE* expression would lead to an increase in *FAMA* as a result of direct regulation of *FAMA* by MUTE [52]. To verify that the MUTE protein accumulates in the gynoecia of mutant *ag* plant precociously, we introgressed the *MUTEpro*:*MUTE-GFP* reporter, which contains upstream and intronic regions of *MUTE* in addition to its coding sequence [16], into *ag-10* and isolated *MUTEpro*:*MUTE-GFP ag-10* and reisolated *MUTEpro*:*MUTE-GFP* in a wild-type background. Through confocal laser microscopy and epifluorescence microscopy, we verified that MUTE-GFP accumulates in valves of stage 11 and early stage 12 *ag-10* gynoecia whereas, in wild-type plants, a strong MUTE-GFP signal was absent from stage 11 gynoecia, largely only visible in the style of early stage 12 gynoecia and valves of late stage 12 (S5 Fig).

### Interaction between AG, SHP, SEP3, and the first intron of *MUTE*

**Fig 4:**
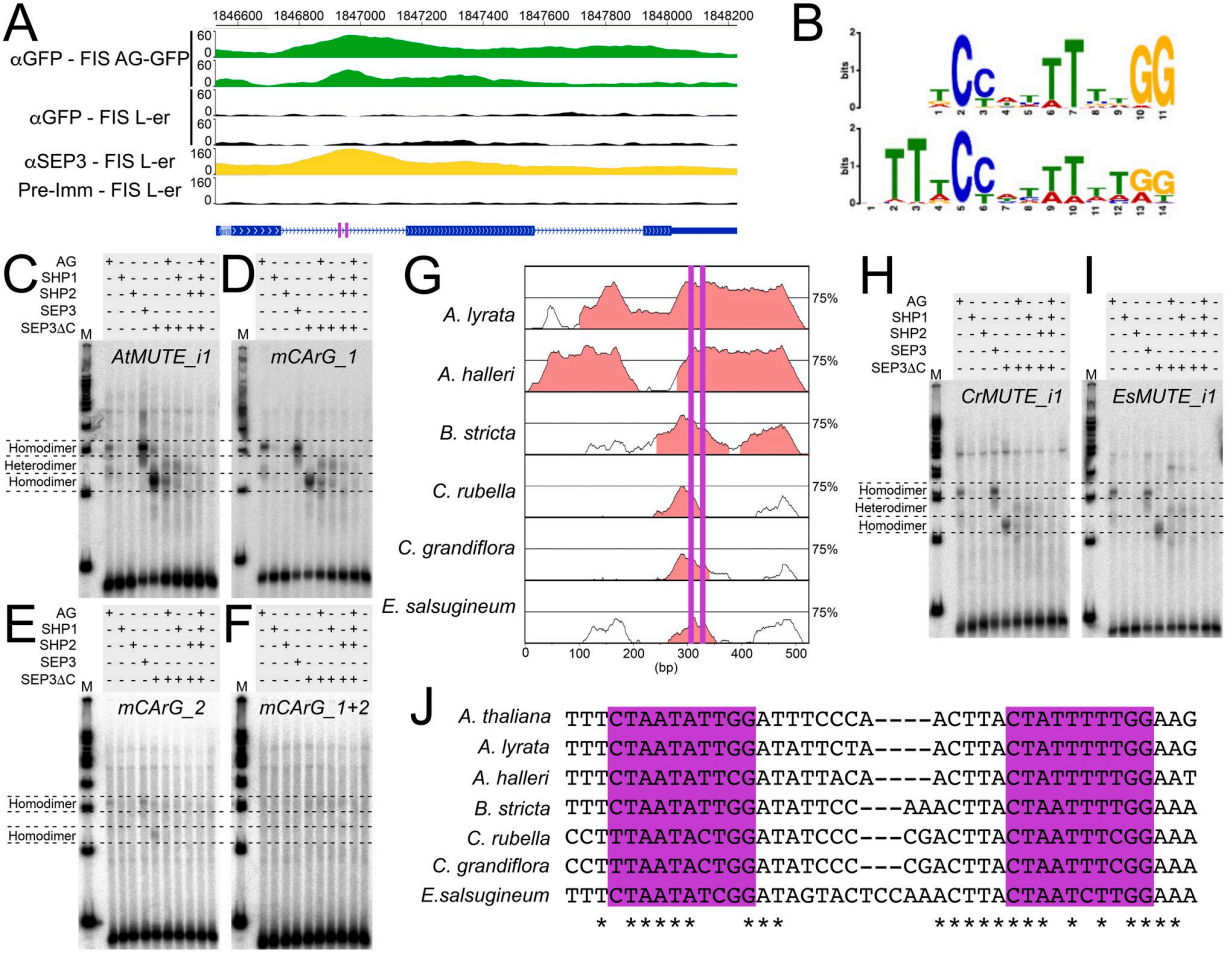
Interaction between AG, SHP1, SHP2, SEP3, and first intron of *MUTE*. (A) Tracks indicating enrichment of sequences in the first intron of *MUTE* from two replicates of ChIP-Seq of AG-GFP (green tracks) and SEP3 (yellow tracks, combined results of two replicates), proteins in the floral induction system (FIS) [41,54]. Corresponding control ChIP-Seq experiments are colored in black. A schematic of the gene structure of *MUTE* blue is below (exon, rectangles; introns, lines). Two CArG motifs were identified within the first intron of *MUTE* (purple rectangles). (B) Binding logos from MEME and STREME analyses of 1421 binding sites identified in a ChIP-Seq of AG [41]. (C-F) Protein-DNA gel shift assays using combinations of AG, SEP3, SEP3ΔC, SHP1, and SHP2 recombinant protein and (C) a wild-type *AtMUTE* probe (*AtMUTE_i1*), (D) a probe where CArG_1 is mutated (*mCArG_1*), (E) a probe where CArG_2 is mutated (*mCArG_2*), and (F) a probe where both CArG motifs are mutated (*mCArG_1+2*). M, molecular weight marker. (G) An mVISTA alignment of the first intron of *MUTE* from various members of the Brassicaceae relative to *A*. *thaliana*. Regions highlighted in salmon have been designated as “Conserved Non-Coding Sequences” by mVISTA. Purple lines indicate the position of each CArG motif. (H-I) Protein-DNA gel shift assays using combinations of AG, SEP3, SEP3ΔC, SHP1, and SHP2 protein and (H) a wild-type *CrMUTE* probe (*CrMUTE_i1*) and (I) a wild-type *EsMUTE* probe (*EsMUTE_i1*). M, molecular weight marker. (J) Sequence alignments using some of the conserved region identified in (B) containing both CArG motifs. Asterisks indicate conserved nucleotide and the two CArG motifs are highlighted by purple rectangles. Unadjusted images for both replicates of the protein-DNA gel shift assays can be found in S10 Fig.

*AG* activity is negatively correlated with stomatal development on gynoecial valves and repression of *AG* activity results in elevated mRNA levels of key stomatal regulators, such as *MUTE* and *FAMA*. To understand whether the regulation of *MUTE* and *FAMA* transcription was directly controlled by AG, we interrogated chromatin immunoprecipitation followed by next-generation sequencing (ChIP-Seq) data available for AG. We found that AG bound to the first intron of *MUTE* (Fig 4A), which was previously identified as a direct target [41]. *MUTE* was also identified as a putative direct target of SEP3 (Fig 4A), which forms heterodimers and quaternary complexes with AG to control floral organ development [29,34,46]. Within the first intron of *MUTE* are two motifs with similarity to CArG motifs, to which these MADS domain transcription factors bind [41,53,54]. Analysis of the genomic sequences bound by AG revealed an enrichment of CArG motifs, which were very similar to the motifs identified in the first intron of *MUTE* (Fig 4B, S5 Table). *MUTE* was also identified as a direct target of the AG-SEP3 complex through sequential immunoprecipitation followed by DNA affinity purification and sequencing experiments (seq-DAP-Seq) (S6 Fig) [55].

We performed gel shift assays to cross-validate these experiments and to determine whether both CArG motifs in the *MUTE* first intron are required for AG and SEP3 binding. We synthesized probes with the same sequence as the region of the *MUTE* first intron that contains the two CArG motifs (*AtMUTE_i1*) and incubated them with the AG and SEP3 proteins (Fig 4C, S6 Table). We also used a truncated version of SEP3 (SEP3ΔC), which has been shown to retain activity [56], to visualize shifts between homodimers of SEP3 and AG-SEP3 heterodimers. We found that incubation of SEP3, SEP3ΔC, or AG individually resulted in the appearance of a single retarded fraction of the DNA probe, suggesting that homodimers of each of these proteins can bind the *MUTE* first intron (Fig 4C). In accordance with the similar size of the full-length proteins, the retarded fractions produced by SEP3 and AG migrated with approximately the same speed, whereas the retarded fraction produced by SEP3ΔC migrated faster, likely due to the reduced protein size. Co-incubation of AG with SEP3ΔC resulted in the appearance of an additional retarded fraction of intermediate electrophoretic mobility, probably corresponding to AG-SEP3ΔC heterodimers. We also incubated the SHP1 and SHP2 paralogs of AG with SEP3ΔC in separate reactions and observed a shifted band when SHP1 was incubated with SEP3ΔC at a height similar to that of the putative AG-SEP3ΔC heterodimers. Only a very weak band was present to suggest that a SHP2-SEP3ΔC heterodimer could bind the *AtMUTE_i1* probe and there was very weak support for SHP1 or SHP2 homodimer binding. Furthermore, incubation of all 4 proteins together did not result in a shift, suggesting that a multimeric complex does not form at the *MUTE* intron (Fig 4C).

Next, we tested whether the CArG motifs present in this region were required for binding. We incubated combinations of the above proteins with probes harboring modifications of the CArG motifs individually (*mCArG_1*, *mCArG_2*) or simultaneously (*mCArG_1*+*2*), which were intended to disrupt protein binding (S6 Table). Similar results to the *AtMUTE_i1* probe were obtained when *mCArG_1* was incubated with the same combinations of proteins as above (Fig 4D). However, incubation with the *mCArG_2* probe abolished the formation of bands corresponding to heterodimer formation. Furthermore, the formation of bands corresponding to homodimers were reduced significantly (Fig 4E). Disruption of both CArG motifs (*mCArG_1+2*) eliminated all shifted bands (Fig 4F). This suggests that CArG_2 interacts with heterodimers and that both CArG motifs can interact with homodimers, although the tested proteins have the highest affinity for CArG_2.

We compared the sequence of the first intron of *A*. *thaliana MUTE* to the sequences of the first intron from orthologs of *MUTE* from other members of the Brassicaceae family (S7 Table). We found that a region of approximately 60 bp was well-conserved, which contained both CArG motifs (Fig 4G). For CArG_2, the AT track in the center of the CArG motif was well maintained in four species. However, a single T to C transition was observed in the AT track of CArG_2 in *Capsella rubella*, *Capsella grandiflora* and *Eutrema salsugineum* (Fig 4J). Therefore, we tested whether AG, SEP3, and/or the SHP proteins could bind to these diverged CArG_2 motifs. We chose the sequences from *C*. *rubella* (Fig 4H), as the CArG_2 motif was identical to *C*. *grandiflora* (Fig 4J), and *E*. *salsugineum* (Fig 4I), and generated probes that encompassed both CArG motifs. We observed near identical results when these probes were incubated with the above proteins when compared to the *AtMUTE_i1* probe (Fig 4H and 4I). This suggests that the interaction between *MUTE*, AG, SEP3, and to a lesser extent SHP1, is conserved among several members of the Brassicaceae.

### Stomatal development on gynoecial valves upon repression of *AG* activity

**Fig 5:**
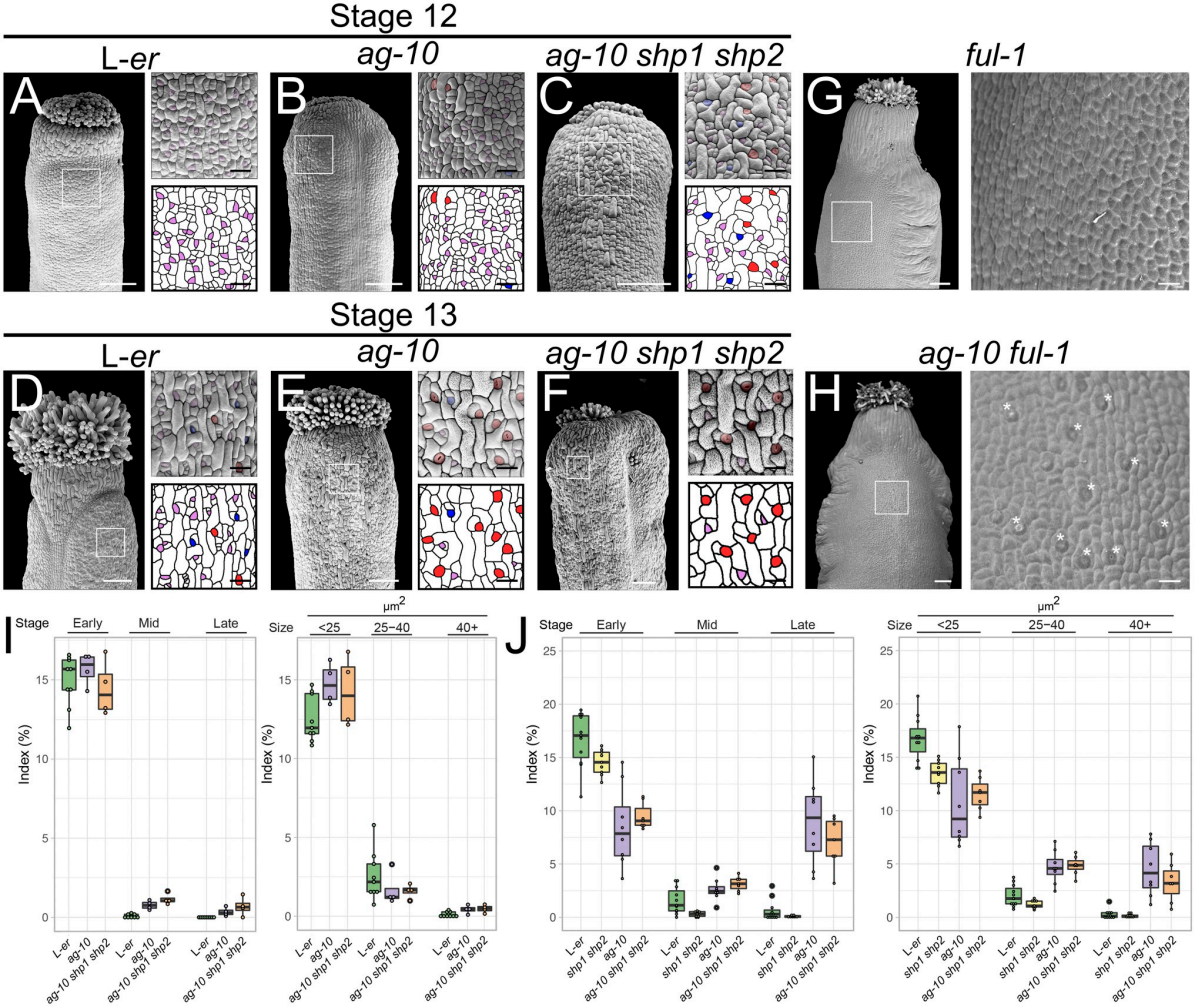
Stomatal development on gynoecial valves in response to reduced *AG* and *SHP* activity. (A-F) Scanning electron micrographs of (A-C) stage 12 and (D-F) stage 13 L-*er*, *ag-10*, and *ag-10 shp1 shp2* gynoecia, as indicated. Purple, blue, and red highlights indicate early, mid, and late-stage stomatal lineage morphology, respectively. (G-H) Scanning electron micrographs of a (G) *ful-1* and (H) *ag-10 ful-1* silique. Asterisks indicate presence of stomata. Scale bars for images of whole gynoecia are 100 μm. Scale bars for magnifications are 20 μm. (I-J) Index of early, mid, and late stomatal lineages based on morphological and cell size analysis of scanning electron micrographs from (I) stage 12 and (J) stage 13 L-er, *shp1 shp2*, *ag-10*, *ag-10 shp1 shp2* gynoecia. Each dot represents an individual sample.

We next asked whether the elevated transcription of *MUTE* and *FAMA* observed in the plants lacking complete *AG* activity was sufficient to modify stomatal formation on the gynoecial valves. Using SEM, we imaged the gynoecia and siliques of wild-type L-*er* and *ag-10* plants (Fig 5A, 5B, 5D, 5E, 5I and 5J). On stage 13 *ag-10* gynoecia, there was a conspicuous abundance of late-stage stomatal cells visible by the presence of symmetric cell divisions and even stomata with open pores (Fig 5E and 5J, S7A and S7B Fig), which were largely absent on L-*er* controls of the same stage (Figs 2B, 5D and 5J). We identified probable stomatal lineage cells from these images and categorized them based on morphology and through cell size analysis (as described in the Materials and methods).

According to morphological analysis, late-stage stomatal lineage cells were absent from stage 12 wild-type gynoecial valves, but were present on stage 12 *ag-10* gynoecial valves (*p*.*adj* = 0.121, pairwise unpaired two-tailed t-test) and were ~4-fold more abundant on *ag-10* gynoecial vales compared to L-er, as determined by cell-size analysis (*p*.*adj* = 0.032, pairwise unpaired two-tailed t-test) (Fig 5A, 5B and 5I, S1 and S2 Tables). At stage 13, mid-staged cells were ~2.4 and ~1.7 times more abundant on *ag-10* gynoecia relative to L-*er* (cell size analysis *p*.*adj* < 10^−5^ and morphological analysis *p*.*adj* = 0.024, pairwise unpaired two-tailed t-tests) and late-stage stomatal cells were 14 to 16 times more abundant on *ag-10* gynoecial valves than L-*er* controls (morphological analysis *p*.*adj* < 10^−7^ and cell size analysis *p*.*adj* < 10^−5^, pairwise unpaired two-tailed t-tests) (Fig 5D, 5E and 5J, S1 and S2 Tables). Concomitantly, we observed a reduction of ~51–64% in the number of early-stage stomatal lineage cells (morphological analysis *p*.*adj* = 10^−6^ and cell size analysis *p*.*adj* = 10^−4^, respectively, pairwise unpaired two-tailed t-tests) (Fig 5D, 5E and 5J, S1 and S2 Tables).

To confirm the stomatal phenotypes we observed of the *ag-10* mutant gynoecia, we also perturbed *AG* activity through dynamic induction of the AG-amiRNA (S7C–S7F Fig). Late-stage cells were 10 to 37 times more abundant on the gynoecia of plants where the AG-amiRNA had been activated relative to control counterparts (morphological analysis *p*.*adj* < 10^−5^ and cell size analysis *p*.*adj* < 10^−4^, respectively, pairwise unpaired two-tailed t-tests) (S7C–S7F Fig, S1 and S2 Tables). Early-stage cells were reduced in abundance to 38–58% on gynoecial valves where the AG-amiRNA was induced relative to uninduced controls (cell size analysis *p*.*adj* = 0.001 and morphological analysis *p*.*adj* < 10^−3^, pairwise unpaired two-tailed t-tests) (S7C–S7F Fig). These data demonstrate that perturbation of *AG* through static mutation (*ag-10*) or through independent dynamic perturbation using the AG-amiRNA could promote the precocious formation of stomatal cells on gynoecial valves.

It has been previously observed that the mutant of another MADS-domain transcription factor-coding gene, *FRUITFULL* (*FUL*), does not bear stomata on the silique [6]. Notably, introgression of the *shp1-1 shp2-1* mutations into the strong *ful-1* mutant results in the recovery of stomata on siliques [57]. We asked whether introduction of the *ag-10* mutant into the *ful-1* background would also be sufficient to rescue the formation of stomata on siliques. We found stomatal formation was partially restored on *ag-10 ful-1* siliques although the stomata were not completely normal in appearance and were not distributed throughout the silique (Fig 5G and 5H). Mean expression of *SHP1* was not reduced (*p* = 0.87, two-tailed paired t-test) and *SHP2* was only mildly reduced 0.87-fold (*p* = 0.12, two-tailed pared t-test) in the *ag-10* background compared to L-*er* (S8A Fig, S3 Table). Therefore, recovery of stomata on *ag-10 ful-1* siliques is probably independent of *SHP* activity. We also assessed the expression of *SPCH*, *MUTE*, and *FAMA* in the gynoecia of stage 12–13 L-*er*, *ful-1*, *ag-10*, and *ful-1 ag-10* plants (S8B–S8D Fig, S3 Table). *SPCH* mRNA levels were reduced in *ful-1* and *ag-10* to ~0.07 and ~0.4-fold (*p*.*adj* = 0.02 in both cases, pairwise paired two-tailed t-tests). *SPCH* mRNA levels were similar to *ful-1* in *ful-1 ag-10* gynoecia (*p*.*adj* = 0.58, pairwise paired two-tailed t-test) indicating that *SPCH* activity was not required to restore the formation of stomatal-like complexes on *ful-1 ag-10* gynoecia (Fig 5G and 5H). *MUTE* mRNA levels were also decreased to 0.04-fold in *ful-1* but increased to 2.64-fold *ag-10*, as previously observed (*p*.*adj* = 0.122 and *p*.*adj* = 0.032, respectively, pairwise paired two-tailed t-tests). In *ful-1 ag-10* double mutants, mean *MUTE* expression increased by 5.8-fold compared to *ful-1* plants although there was variation associated with this measurement (*p*.*adj* = 0.194, pairwise paired two-tailed t-test) (S8C Fig). Similarly, mean *FAMA* mRNA levels were decreased to 0.23-fold in *ful-1* but increased to 2.59-fold *ag-10* (*p*.*adj* = 0.13 in both cases, pairwise paired two-tailed t-tests). In *ful-1 ag-10* gynoecia *FAMA* levels were increased by ~5.4-fold compared to *ful-1* (*p*.*adj* = 0.1, pairwise paired two-tailed t-test) (S8D Fig). Both *MUTE* and *FAMA* levels were recovered towards levels similar to L-*er* in *ag-10 ful-1* (*p*.*adj* = 0.404 and *p*.*adj* = 0.64, respectively, pairwise paired two-tailed t-tests) in comparison to the single mutants (S8C and S8D Fig). These data are consistent with the idea that the genetic pathway controlled by *AG* in the regulation of stomatal development converges on *MUTE* and demonstrate that AG is required to suppress stomatal development in *ful-1* mutants during gynoecium development.

### Comparative activity of *AG* and *SHPs* during stomatal development

To understand more about the redundancy between *AG* and the *SHP* genes during stomatal development, we generated an *ag-10 shp1 shp2* triple mutant and examined the stomatal lineage indices on gynoecial and silique valves (S9A–S9D Fig). Assessment of the data through both morphological and cell size analysis, we found very little difference between the abundance of mid-stage and late-stage cells on *ag-10 shp1 shp2* gynoecia relative to *ag-10* at stage 12 or 13 (Fig 5B, 5C, 5E, 5F, 5I and 5J, S1 and S2 Tables). Therefore, the introduction of the *shp1 shp2* alleles do not appear to be able to enhance the stomatal phenotype of the *ag-10* mutant.

**Fig 6:**
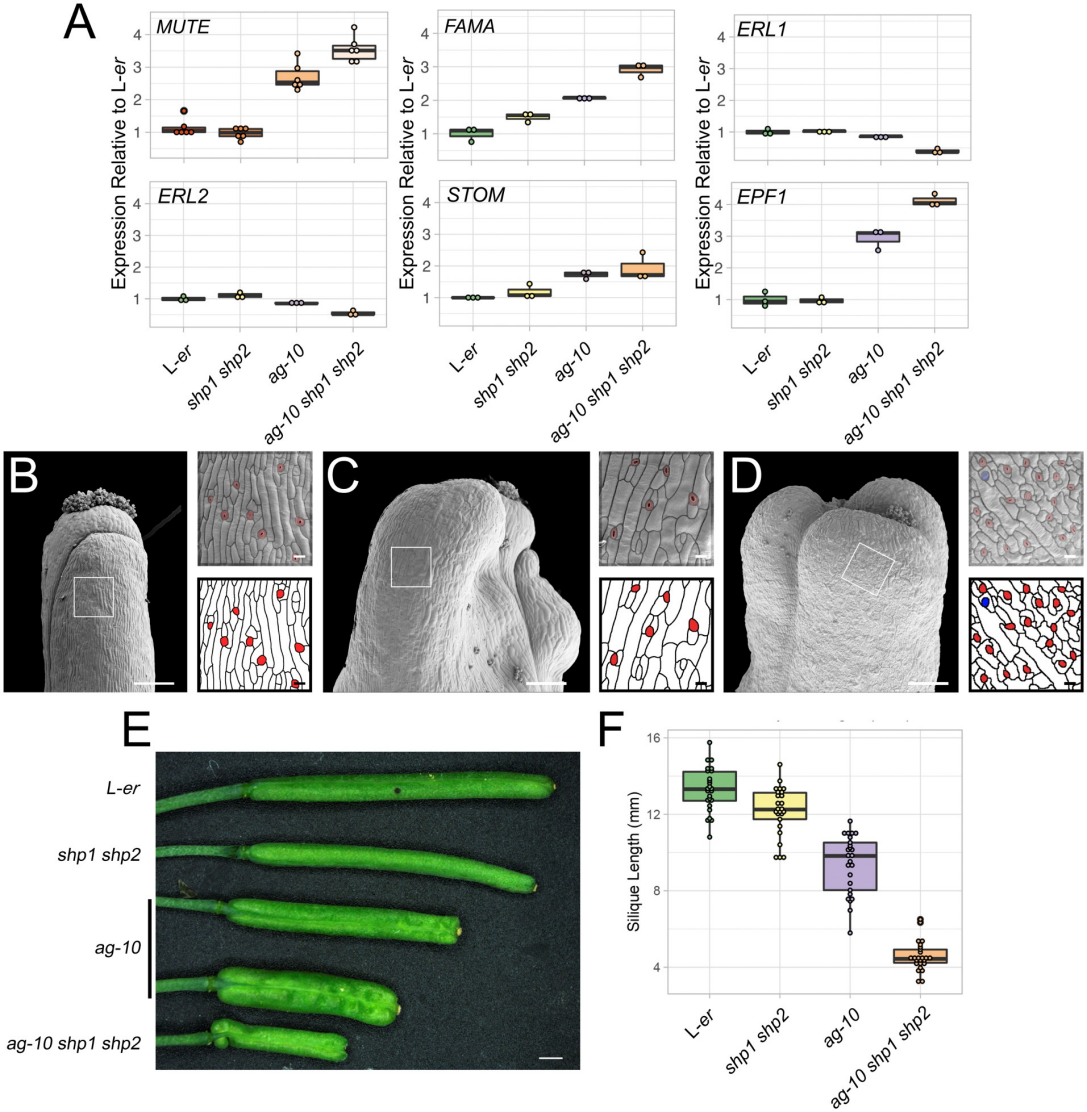
Redundancy between *AG* and *SHP1/2* during gynoecium and silique development. (A) Levels of *MUTE*, *FAMA*, *ERL1*, *ERL2*, *STOM*, and *EPF1* mRNAs as determined by RT-qPCR, in L-*er*, *shp1 shp2*, *ag-10*, *ag-10 shp1 shp2* stage 10–13 gynoecia relative to average of L-*er* samples. Each dot represents the technical mean of an individual independent biological replicate. (B-D) Scanning electron micrographs of mature siliques of (B) L-*er*, (C) *ag-10*, and (D) *ag-10 shp1 shp2*. Purple, blue, and red highlights indicate early, mid, and late-stage stomatal lineage morphology. Scale bars for images of whole gynoecia are 200 μm. Scale bars for magnifications are 20 μm. (E) Mature siliques of L-*er*, *shp1 shp2*, *ag-10*, *ag-10 shp1 shp2*. Scale is 1 mm. (F) Length of L-*er*, *shp1 shp2*, *ag-10*, *ag-10 shp1 shp2* mature siliques.

We also examined the mRNA levels of *SPCH*, *MUTE*, and *FAMA* in this *ag-10 shp1 shp2* triple mutant background in comparison to parental genotypes (Fig 6A, S9E Fig, S3 Table). Mean *SPCH* levels were reduced in the *ag-10 shp1 shp2* triple mutant relative to *ag-10* to ~0.79 fold, although the variation in expression was quite high (*p*.*adj* = 0.55, pairwise two-tailed t-test) (S9E Fig, S3 Table). Mean *MUTE* mRNA levels were elevated in the *ag-10 shp1 shp2* triple mutant relative to *ag-10* to ~1.33 fold (*p*.*adj* = 0.012, pairwise two-tailed t-test). *FAMA* mRNA levels were also elevated to ~1.4 fold in the *ag-10 shp1 shp2* triple mutant relative to *ag-10* (*p*.*adj* = 0.022, pairwise two-tailed t-test, Fig 6A, S3 Table). In contrast, levels of *SPCH*, *MUTE*, and *FAMA* mRNAs were not as strongly affected in the *shp1-1 shp2-1* double mutant background (Fig 6A, S3 Table). We concluded that the SHP proteins probably suppress the expression of *MUTE* in parallel with AG, however, this increased expression does not appear to be sufficient to enhance the phenotype of *ag-10 shp1 shp2* relative to *ag-10*.

Two other genes that are associated with AG binding are also involved in stomatal development, *ERL1* and *ERL2* [41]. These genes encode receptor-like kinases that control stomatal patterning in concert with TMM [20]. Ligands, such as STOMAGEN (STOM) and EPF1, bind to complexes of TMM, ER, ERL1, and ERL2, to maintain stomatal spacing and density [15]. Therefore, we tested the mRNA levels of these genes in response to reduced *AG* and *SHP* activity. *ERL1* and *ERL2* expression were mildly decreased in *ag-10*, however, their mRNA levels were reduced to ~0.4 (*p*.*adj* = 0.038, pairwise two-tailed t-test) and ~0.5-fold (*p*.*adj* = 0.009, pairwise two-tailed t-test) in the triple *ag-10 shp1 shp2* mutant relative to L-*er*, respectively (Fig 6A, S3 Table). *EPF1* mRNA levels were elevated in both *ag-10* and the triple mutant to ~3-fold (*p*.*adj* = 0.009, pairwise two-tailed t-test) and ~4.3-fold (*p*.*adj* = 0.009, pairwise two-tailed t-test), respectively (Fig 6A, S3 Table). *EPF1* levels were elevated by ~1.4-fold in the triple mutant relative to *ag-10* (*p*.*adj* = 0.03, pairwise two-tailed t-test) (Fig 6A, S3 Table). Similarly, mean *STOM* expression was elevated in *ag-10* and the triple mutant by ~1.7 (*p*.*adj* = 0.06, pairwise two-tailed t-test) and ~1.9-fold (*p*.*adj* = 0.14, pairwise two-tailed t-test) (Fig 6A, S3 Table). In contrast to these observations, mean *TMM* mRNA levels were not changed in *ag-10* or the *ag-10 shp1 shp2* triple mutant backgrounds (*p* = 0.39, two-way ANOVA) (S9F Fig, S3 Table). Furthermore, there was little change in the expression of these genes in the *shp1 shp2* mutant background (Fig 6A, S7E and S7F Fig, S3 Table).

Although the accumulation of *ERL1*, *ERL2*, *STOM*, and *EPF1* mRNAs were changed in the *ag-10* and/or the *ag-10 shp1 shp2* mutant backgrounds, we did not observe stomatal clustering in the samples in which *AG* or *SHP* were perturbed (Fig 6B–6D). This suggests that either the observed changes are not substantial enough to modify patterning or that an initial change in gene expression mediated by *AG* and/or *SHP* was buffered by the regulatory network to maintain patterning. Notably, combinations of *er*, *erl1*, and *erl2* mutations resulted in a reduction of silique length [58], which is similar to the reduction of silique length observed for *ag-10 shp1 shp2* plants relative to parental genotypes and wild-type (Fig 6E and 6F, S8 Table). Therefore, it is likely that AG-mediated control of *ERL1* and *ERL2* expression is related only to gynoecial growth rather than stomatal patterning.

## Discussion

The progression of stomatal development on the gynoecium/silique has not been previously described. Here, we show that stomatal development initiates on the gynoecial valves between late stage 11 and early stage 12 (Fig 2B–2E, S1 and S2 Tables). Stomatal initiation on the gynoecium correlates with the increased expression of stomatal bHLH transcription factors during this period (Figs 1 and 2I). Most stomates do not reach maturity until after fertilization, although their maturation does not depend on fertilization (S2C and S2D Fig). This is reminiscent of other differentiation processes, such as the continued increase in chlorophyll concentration in the gynoecia of emasculated pistils (i.e. unfertilized) [59], which also temporally correlate with, but are not dependent on, fertilization [60]. Given that fertilization does not control the onset or progression of stomatal development on gynoecial valves, we searched for an alternative mechanism.

**Fig 7:**
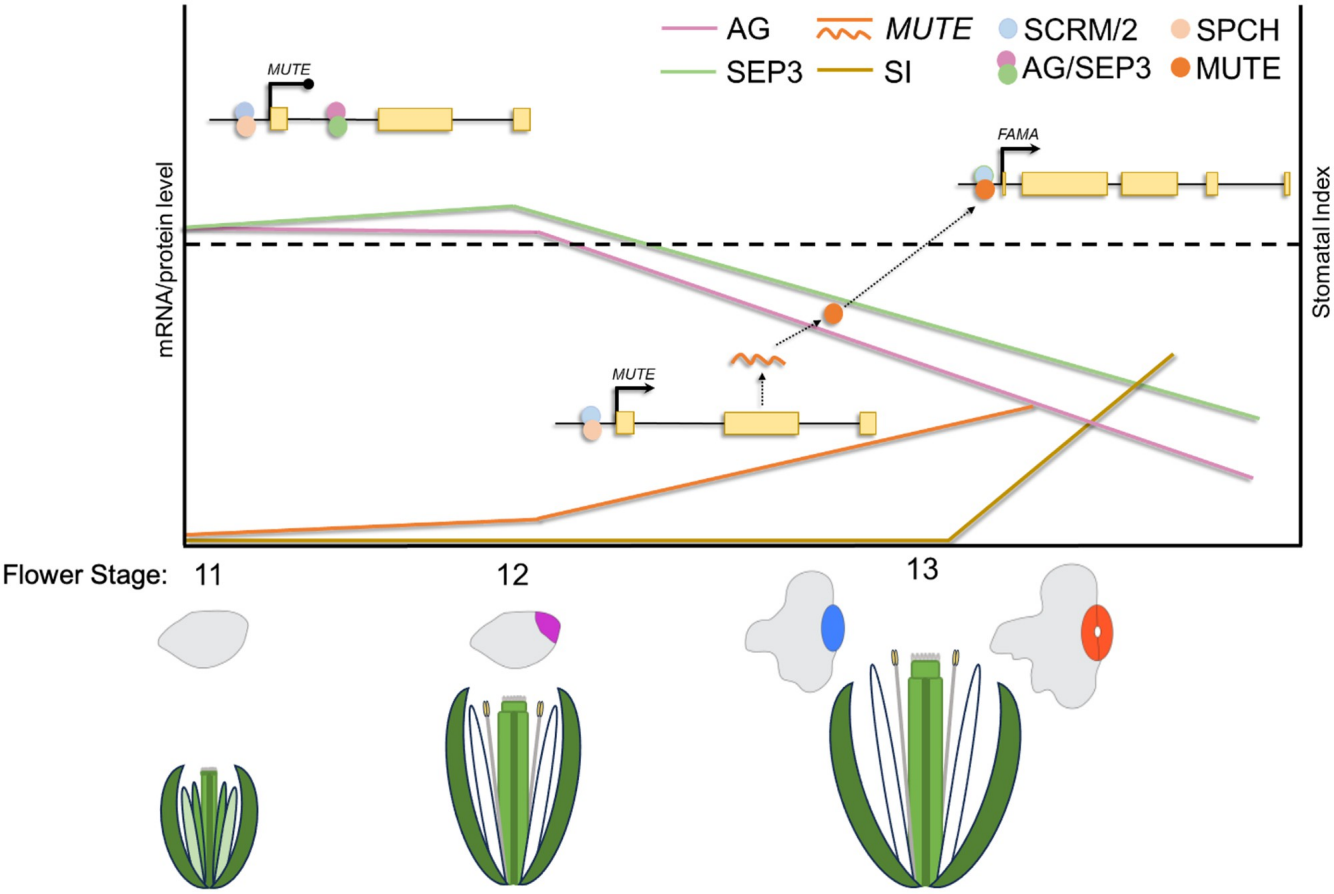
Model describing regulation of *MUTE* expression before and after fertilization. Before fertilization (i.e., before stage 13) a heterodimer of AGAMOUS (AG) and SEP3 (pink and green circles, respectively) are bound to the first intron of *MUTE*, which act to repress its transcription (roundhead line). At the same time, dimers of SPEECHLESS (SPCH, light orange circles) and SCREAM (SCRM)/SCRM2 (light blue circles) are likely bound to the promoter of *MUTE*. As AG and SEP3 protein levels (pink and green lines, respectively) decrease in the valve they reach a critical threshold (dotted line) where they no longer efficiently repress *MUTE* transcription. The absence of the AG-SEP3 dimer allows the SPCH-SCRM/2 complex to promote *MUTE* transcription (arrowhead). *MUTE* mRNA levels begin to increase (orange line) and after translation of the *MUTE* mRNA into protein (wavy orange line and circle, respectively), a complex of MUTE-SCRM/2 promotes the expression of *FAMA* so that mature stomatal complexes begin to form to coincide with fertilization (stage 13), represented by the stomatal index (SI, brown line). Activation of *MUTE* expression by SPCH has not been demonstrated, although SPCH binding to the *MUTE* promoter has been detected [61]. SPCH may promote the expression other genes whose products are responsible for directly promoting *MUTE* expression [61,62]. Gene bodies are depicted by solid black lines (up and downstream regions, introns) and yellow rectangles (exons). Cartoons of stomatal lineage and flower development stages are depicted below.

The floral organ identity gene *AG* has been implicated previously in the differentiation of the gynoecial valve and in the suppression of leaf-like traits [35,41]. Here, we present evidence that AG forms a complex with SEP3 to suppress the formation of stomata (Fig 5), which are also typical of leaves, until the point of anthesis when their expression dissipates from gynoecial valves (Fig 2F–2H and S3 Fig). AG and SEP3 accomplish this by directly repressing the transcription of *MUTE*, which encodes a key bHLH transcription factor required for stomatal development (Figs 3 and 4, S5 Fig) [52]. *MUTE* mRNA levels are elevated in the gynoecia of plants with reduced *AG* activity. In contrast, *SPCH* mRNA levels are decreased at certain time-points when *AG* activity is decreased (Fig 3C and 3F), suggesting that *AG* can promote *SPCH* expression although there is no indication that AG performs this action directly. The expression of *FAMA*, which is directly promoted by MUTE [52], is elevated when *AG* activity is perturbed (Fig 3). By visualizing *FAMA* expression using a transgenic reporter line [51], we showed that repression of *AG* activity does not release *MUTE* or *FAMA* expression throughout the epidermis of the gynoecium but only in what are likely stomatal lineage cells (Fig 3G–3J, S5 Fig). This suggests that SPCH facilitates *MUTE* expression in a similar manner as during leaf development and that AG counteracts this SPCH activity in stomatal lineage cells. *MUTE* expression gradually increases in stomatal lineage cells once AG-SEP3 protein levels reduce below a critical threshold (Fig 7), enabling *MUTE* transcription to be fully activated. Therefore, stomatal lineage progression, but not entry, is modified by AG. Furthermore, this suggests that the SPCH-SCRM/2 complex [61], or another complex [62,63], is ‘poised’ to promote *MUTE* transcription once activity of the AG-SEP3 complex has diminished.

AG and SEP3 bind to the first intron of *MUTE*, which was previously demonstrated by ChIP-Seq and seq-DAP-seq experiments [41,54,55]. The bound regions contain two CArG motifs and we showed that each CArG motif influences binding in a different way (Fig 4). We confirmed that both homodimers and heterodimers of AG and SEP3 can bind to the first intron of *MUTE*. We also showed that heterodimers of SHP1-SEP3 bind to the same region. Heterodimer binding was dependent on the presence of only one of these CArG motifs (CArG_2), whereas homodimers did not fully depend on the presence of CArG_2 although homodimer affinity was reduced in its absence. Mutagenesis of both CArG motifs, however, abolished binding of both homodimers and heterodimers (Fig 4C–4F). AG-SEP3 quartets are required to maintain floral meristem determinacy and floral organ identity, however, the role of tetramers during stomatal differentiation on the gynoecium is unclear [46,64]. Here, we did not observe the formation of higher order complexes with the generated probes suggesting that homodimers or heterodimers are sufficient to suppress *MUTE* expression though further investigation is required to clarify this. Interestingly, the CArG_1 and CArG_2 motifs that AG and SEP3 bind to (Fig 4, S6 Fig) are also conserved in several members of the Brassicaceae (Fig 4G and 4J, S7 Fig). This data, combined with evidence that *MUTE* is differentially expressed in gynoecia with reduced *AG* activity (Fig 3A, 3B, 3D and 3F, S5 Fig), strongly suggests that the CArG motifs identified are functional. To confirm this, further studies including mutagenesis of these *cis*-regulatory elements is required.

*AG*, *SHP1*, and *SHP2* are known to act redundantly during floral organ specification and trichome suppression and the corresponding proteins are biochemically similar in their activities [35,65]. We found evidence for redundancy between these genes during silique growth, which is likely because of their direct promotion of *ERL1* and *ERL2* expression in an unequally redundant manner by AG, SHP1, and SHP2 (Fig 6A) [66]. Other genes involved in stomatal formation also appear to be unequally redundantly controlled by AG and SHP1/2 expression including *MUTE*, *FAMA*, and *EPF1* (Fig 6A). Heterodimers of SHP1-SEP3 and, to a lesser extent, SHP2-SEP3 were also capable of binding to the *MUTE* first intron (Fig 4). However, introduction of *shp1 shp2* mutant alleles into *ag-10* could not reproducibly enhance the stomatal phenotype of *ag-10* (Fig 5A–5F, 5I and 5J, S1 and S2 Tables). In contrast, introduction of *shp1 shp2* into the *ful-1* mutant background could partially restore stomatal formation on *ful-1* mutant gynoecia [57], similar the introduction of *ag-10* into *ful-1* (Fig 5G and 5H). Furthermore, ectopic expression of both *SHP1* and *SHP2* can suppress the formation of stomata on silique valves [67]. Therefore, we conclude that there is unequal redundancy between SHP1/2 and AG in the regulation of *MUTE* expression [66], with AG playing the predominant role, but increased expression of *MUTE* in *ag-10 shp1 shp2* is not sufficient to substantially enhance the stomatal phenotype of *ag-10* gynoecia.

Interestingly, stomata that form on the style are not impacted by the repression or developmental expression of *AG* (S1C Fig). This may be because AG lacks the appropriate interaction partners in style tissue to suppress *MUTE* expression or because the chromatin landscape inhibits AG-mediated suppression of *MUTE*. We propose that AG acts as a timer to control the emergence of stomata on the developing gynoecium/silique valves in *A*. *thaliana* (Fig 7), which is an important step in the establishment of silique photosynthesis. The conserved CArG motifs in the first intron of *MUTE* and the ability of AG, SEP3, and the SHPs to bind even slightly diverged sequences in *C*. *rubella* and *E*. *salsugineum* suggests that this function is conserved among several members of the Brassicaceae (Fig 4H and 4I). The correlation between stomatal development and fertilization suggests that atmospheric carbon assimilation by the silique stomata is used during silique photosynthesis to support seed development, particularly in terms of seed oil biosynthesis. Oils are stored in the seeds and mobilized during germination and early seedling development before autotrophic growth is established [68]. Energy derived from silique photosynthesis supplements the accumulation of storage oils in the seeds in a variety of species including *A*. *thaliana* and *Brassica napus* [7–10].

The timing of stomatal formation on the silique valves may have been selected for a variety of reasons. Pre-anthesis formation of stomata may be disadvantageous due to selective pressure by pathogens [69]. Pathogens can penetrate the plant through stomata and photosynthetic activity would probably be low in pre-anthesis gynoecia due to the shading effect of sepals. Furthermore, transpiration within a closed flower bud is unlikely to be particularly effective. These factors may have resulted in selection for stomatal formation on the gynoecium/silique to coincide with flower bud opening. It is also possible that the timing of stomatal progression on gynoecial valves differs between species as stomatal development in *A*. *thaliana* had not been completed even once siliques were exposed to light (e.g. stage 15–16, S1 and S2 Tables). Therefore, there appears to be opportunity to initiate stomatal development at an earlier stage so that a higher number of active stomatal complexes are present once the silique is exposed to light. If so, this may result from the modulation of AG function caused by divergence of the *cis* regulatory elements AG binds to. Assessing the correlation between stomatal development and fertilization in other species will partially address these questions.

## Materials and methods

### Plant growth and materials

Plants were grown on soil under cool white light at ~20°C in 16:8 h light:dark conditions. Plant lines used in this study are listed in S9 Table. Flower development stages were previously described [1].

### Genotyping

Genotyping PCRs were performed with genomic DNA extracted as previously described [70]. Tissue was disrupted using a TissueLyser II (Qiagen). Primers used in this study are listed in S10 Table. The genotyping of *ag-10* and *ful-1* was performed as previously described [50,71].

### Confocal microscopy

A Zeiss Laser Scanning Microscope-780, 880, or Olympus FluoView1000 laser scanning microscope was used to generate the fluorescent images. Settings were optimized to visualize GFP, YFP (laser wavelength, 488 nm; detection wavelength, 493 to 598 nm) or chlorophyll (laser wavelength, 545 nm; detection wavelength, 604 to 694 nm). All direct comparisons of images were performed with the same settings. Maximum intensity projections were generated from z-stacks of multiple tiles to visualize the entire apical region of the gynoecium/silique. Samples were mounted on a glass slide with 1% low-melt agarose (Bio-Rad) and submerged in de-ionized water or mounted in 30% glycerol and a cover slip was placed on top and sealed with nail varnish.

### Fluorescence microscopy

Samples were processed through the ClearSee protocol [72] to remove chlorophyll without inactivating fluorescent proteins. Gynoecia were harvested in 4% paraformaldehyde (18501, Mason Technology) on ice. Samples were then incubated in a vacuum at -700 mbar for 30 min at room temperature, the vacuum was released and then applied again for 30 min. The vacuum was then released, and samples were incubated at room temperature for 60 min. The 4% paraformaldehyde was removed, and the samples were washed with 1x PBS (Oxoid X6571f) three times. The samples were then submerged in ClearSee solution (100 g/L xylitol powder 493718, Fluorochem; 150 g/L sodium deoxycholate F545118, Fluka; 250 g/L Urea 51459, Fluka) and incubated in the dark at room temperature for 3 d. Then samples were mounted in 30% glycerol on a glass slide and a cover slip was placed on top and sealed with nail varnish. The samples were then imaged with an Olympus IX81 fluorescence microscope (Excitation 488 nm and detected with a 495/10 nm bandpass filter).

### Use of publicly available genomics data

M-values for the expression of all genes detected during flower development were previously generated [42]. Transcripts per million for each biological replicate and time-point for laser-microdissection RNA-Seq were generously shared with us by Dr. Annette Becker and Clemens Rössner (Justus-Liebig Universität Giessen, Germany) [43]. ChIP-Seq peaks were visualized using online software at ChIP-Hub (https://biobigdata.nju.edu.cn/ChIPHub/) [73]. Seq-DAP-seq sequences were downloaded from the genome browser (https://genome.ucsc.edu/s/ArnaudStigliani/MADS) [55].

### Scanning electron microscopy

Samples were fixed and processed for SEM as previously described [74]. Briefly, samples were harvested in 1x PBS (P4417-100TAB, Sigma-Aldrich) at room temperature. The PBS was then replaced with a 4% solution of 50% glutaraldehyde (4995.1, Roth) in 1 x PBS and incubated overnight at 4°C. A dehydration series was then performed using freshly prepared solutions of 30%, 50%, 70%, and 90% ethanol (v/v) at room temperature for 30 min each while being gently rocked. Samples were then incubated in 100% ethanol for 60 min at room temperature. Ethanol was then replaced with 100% ethanol and placed at 4°C. A Leica CPD300 or Quorum K850 were used for critical point drying and samples were coated with gold using an Emitech SC 7640 (Polaron) or Quorum Q150T sputter coater. Samples were imaged using a Hitachi TM 4000Plus or a Supra 40VP scanning electron microscope (Zeiss).

### Stomatal image analysis

Images were imported into Adobe Photoshop and tracings of the cell margins were drawn by hand. Cells of the stomatal lineage were then differentially colored based on the presence of an asymmetric cell division and/or presumed early stage GMCs (Early stage), the rounding of a cell similar to a GMC (Mid stage), and the presence of a symmetric cleavage (Late stage). Colored cells and total cell numbers were counted with Fiji using *Color Threshold* modifications combined with the *Analyze Particles* function [75].

For the cell size analysis, the same stomatal lineage cells identified through morphological assessment were measured using *Color Threshold* modifications combined with the *Analyze Particles* function following a scale adjustment based on the scanning electron micrograph scale. Cell sizes were assigned to bins of 5 μm^2^ increments to assist with selecting cut-offs to define stomatal lineage progression. Cell size cut-offs for different phases of the stomatal lineage were based on assessment of the progression of wild-type stage 12 and stage 13 L-*er* gynoecia to facilitate direct comparisons with the mutant data. The mean size of early-stage cells from stage 12 and 13 was calculated to be 15.33 μm^2^ with a standard deviation of 7.72 μm^2^. To make the cut-off for early-stage cells, we added 1 standard deviation to the mean (i.e. 23.05 μm^2^, rounded up to 25 μm^2^ for use with the binned data) and defined cells smaller than 25 μm^2^ as early-stage cells. Notably, the selected cut-off value for early-stage cells is very similar to the size of meristemoids when they begin differentiation in leaves, based on both experimental (23.2 μm^2^) and modelling approaches (22 μm^2^) [76]. The mean area of late-stage cells from stage 13 gynoecia was calculated at 31.13 μm^2^ with a standard deviation of 9.02 μm^2^. To make the cut-off for late-stage cells, we added 1 standard deviation to the mean (i.e. 40.15 μm^2^, rounded to 40 μm^2^ for use with the binned data) and defined cells larger than 40 μm^2^ as late-stage cells. As such, cells between 25–40 μm^2^ were defined as mid-stage cells. Data underlying figures representing these experiments are provided in S1 Data.

### Chemical treatments

A solution containing 10 μM dexamethasone (DEX; Sigma) and 0.015% Silwet L-77 in deionized water was applied liberally to the inflorescences of plants with a Pasteur pipette. Mock treatments contained all the same components without dexamethasone. Ethanol vapor treatments were performed by sealing the plants in containers (18 cm x 32 cm x 50 cm) with clear plastic lids and two 50 mL tubes that each contained 10 mL of 100% ethanol for 6 h.

### Reverse transcription quantitative PCR

Samples were harvested on liquid nitrogen and stored at -80°C until processed. Harvesting of gynoecia between stages 10–13 was done under a stereomicroscope from the primary inflorescences of 15–20 plants. Only gynoecia, where the majority of the stigmatic tissue was clear of pollen (i.e., largely unfertilized), were harvested. Harvesting of stage 13 gynoecia from the *AG-amiRNA*^*i*^ line for the time-course (Fig 3F) was done by initially removing all flowers and siliques that were beyond stage 13. Then flowers at anthesis were harvested for the 0 d time-point. Plants were treated with DEX and 1 d later, 2–3 flowers from the same plants at anthesis were harvested on liquid nitrogen. This continued until 7 d after the treatment from 15–20 plants.

Samples were disrupted using a TissueLyser II (Qiagen) with adapters that were kept at -80°C. Total RNA was extracted from the samples using an RNeasy Plant Mini kit (Qiagen), a Spectrum Plant Total RNA kit (Sigma-Aldrich) or a GeneJET Plant RNA Purification Kit (Thermo Fisher Scientific). Removal of genomic DNA was performed with the Turbo DNA-free Kit (Invitrogen), the On-Column DNase I Digestion set (Sigma-Aldrich) or DNaseI (Thermo Fisher Scientific). Reverse transcription was performed with the SuperScript IV First-Strand Synthesis System (Invitrogen) or RevertAid H Minus Reverse Transcriptase (Thermo Fisher Scientific). Quantitative PCR was performed with either iQ SYBR green (Bio-Rad) or PowerUp SYBR Green (Thermo Fisher Scientific) using a Lightcycler 480 (Roche) or Stratagene Mx3005 (Agilent Technologies). At least 2 technical measurements for each biological replicate were made and the mean of these technical replicates was used to represent each biological replicate. The number of independent biological replicates for each RT-qPCR experiment is indicated in S3 Table. Melting curves were obtained for the reactions, revealing single peak melting curves for the amplified products. The amplification data were analyzed using the second derivative maximum method, and resulting values were converted into relative expression values using the comparative cycle threshold method [77]. Primers used in this study are listed in S10 Table. Data underlying figures representing these experiments are provided in S1 Data.

### Motif enrichment analysis

The 1421 sequences identified as bound by AG were downloaded from www.araport.org [41,78]. These sequences were uploaded to the MEME-ChIP interface on meme-suite.org [79,80]. The settings applied and motifs identified are summarized in S5 Table.

### Genomic sequence alignments

The genomic and inferred amino acid sequences of *MUTE* were downloaded from The Arabidopsis Information Resource (www.arabidopsis.org) [78]. Orthologs of *AtMUTE* were previously identified in *Arabidopsis lyrata*, *Capsella rubella*, and *Eutrema salsugineum* (formerly *Thellungiella halophila*) [81]. We identified putative orthologs in *Capsella grandiflora*, *Arabidopsis halleri*, and *Boechera stricta* by using the TBLASTN functionality in Phytozome (https://phytozome-next.jgi.doe.gov/) with the AtMUTE amino acid sequence against the above genomes [82]. We found one clear candidate in each genome, which included the same orthologs identified previously [81]. The TBLASTN search results are presented in S7 Table. Sequence alignments were performed with mVISTA (https://genome.lbl.gov/vista/mvista/submit.shtml) using standard settings [83].

### Statistical analysis

Some recommendations from *The American Statistician* are implemented here [84]. The term “statistically significant” has been avoided and *p*-values have been reported as continuous where possible. Although grouping data based on statistical thresholds is not recommended [84], a grouping threshold is provided in supplemental tables (S1–S10 Tables) as a helpful guide, but results are not interpreted on the basis of these groupings. Data were analyzed using the R studio software [85]. For multiple comparisons, data were analyzed using a one-way analysis of variance (ANOVA). Post hoc tests were employed if a significant result was obtained from the above tests (as described in S1, S2, S3, S4 and S8 Tables). The base function pairwise.t.test was used following an ANOVA test, with analysis being paired or unpaired depending on the experimental design. Adjustments for multiple corrections were performed in each case using the Benjamini–Hochberg method [86]. For the box plots, the box encapsulates the 25th to 75th percentile, and the error bars encapsulate the 10th and 90th percentiles. The horizontal line running through the box indicates the median, and each point represents an individual plant.

### Gel shift assays

Electrophoretic mobility shift assays were performed essentially as described previously [56]. DNA probes were ordered as single stranded oligo, annealed, and radioactively labelled with [α-P^32^] dATP by a Klenow fill-in reaction of 5`-overhangs. Probe sequences are shown in S6 Table. Proteins were produced *in vitro* via the TNT SP6 Quick Coupled Transcription/Translation System (Promega) following manufacturer’s instructions. Plasmids for *in vitro* transcription/translation of SEP3 and SEP3ΔC have been generated previously [56]. For generation of the plasmids for *in vitro* transcription/translation of AG, SHP1, and SHP2, coding sequences of *AG* (X53579.1), *SHP1 (NM_115740*.*3)*, and *SHP2* (NM_129844.5) were synthesized via the GeneArt gene synthesis service (Thermo Fisher Scientific) and cloned into pTNT (Promega) using *Eco*RI and *Sal*I restriction sites. The composition of the protein-DNA binding reaction buffer was essentially as described previously with final concentrations of 1.6 mM EDTA, 10.3 mM HEPES, 1 mM DTT, 1.3 mM spermidine hydrochloride, 33.3 ng μL^-1^ Poly dI/dC, 2.5% CHAPS, 4.3% glycerol, and 6 μg μL^-1^ BSA [87]. For each binding reaction 2 μL of *in vitro* transcribed/translated protein were co-incubated with 0.1 ng of radioactively labelled DNA probe in a total volume 12 μL. Binding reactions were incubated overnight at 4°C and loaded on a polyacrylamide (5% acrylamide, 0.1725% bisacrylamide) 0.5× TBE gel. The gel was run at room temperature with 0.5× TBE buffer for 3 h at 7.5 V cm^–1^, and afterwards vacuum-dried and exposed onto a phosphorimaging screen to quantify signal intensities. For size comparison a radioactively labelled DNA ladder (100 bp DNA ladder, New England Biolabs) was applied. Original gels are shown in S10 Fig.

## Supporting information

S1 Fig Overview of stomatal and gynoecium development and the presence of stomata on floral organs. (A) A scanning electron micrograph of a gynoecium at stage 13 (anthesis) with the valves (green), replum (red), style (blue) and stigma (yellow) false colored. (B) Simplified model of stomatal development on leaves. The bHLH transcription factors, SPEECHLESS (SPCH), MUTE, FAMA, SCREAM (SCRM) and SCRM2 coordinate the progression of stomatal development. SPCH is subject to post-translational regulation by a mitogen activated protein kinase (MAPK) cascade, which is activated by receptors, such as TOO MANY MOUTHS (TMM), ERECTA (ER) and ER-LIKEs (ERLs). The secreted peptides, EPIDERMAL PATTERNING FACTOR1 (EPF1) and EPF2 bind to these receptors to activate them at different stages of stomatal development. EPF-LIKE9/STOMAGEN (STOM) competes with EPF1/EPF2 binding to suppress activation of the receptors. The dotted line from the ERL1-TMM complex to the SCRM/2-MUTE complex indicates an unknown mechanism of repression. (C-F) Scanning electron micrographs of (C) a stage 12 gynoecium, (D) a stage 10 abaxial sepal, (E) a stage 11 abaxial anther, and (F) a stage >17 silique. Asterisks indicate presence of stomatal lineage cells. Red box in (F) highlights the size of style tissue in comparison to valve tissue, both of which bear stomata. Scale in (C) 100 μm, (D-E) 20 μm, and (F) 200 μm.
(TIF)

S2 Fig Progression of stomatal lineage on wild-type L-*er* gynoecia and siliques. (A-B) The progression of the stomatal lineage on wild-type gynoecia and siliques as determined by (A) morphological assessment and (B) by cell size analysis. (C-D) Scanning electron micrographs of (C) a gynoecium from an emasculated flower 5 days after anthesis, (D) a magnification of the valve in (C). Scale in (C) 200 μm, (D) 20 μm.
(TIF)

S3 Fig Confocal imaging of AG-GFP in late-stage gynoecia. (A-C) Maximum intensity projections of stitched confocal laser scanning z-stack micrographs of (A) early stage 12, (B) late stage 12, and (C) stage 13 gynoecia from *AGpro*:*AG-GFP ag-1* plants. Arrowheads indicate accumulation of AG-GFP in the replum. Scale is 100 μm.
(TIF)

S4 Fig Confocal imaging of *FAMApro*:*2xYFP* in the *AG-amiRNA*^*i*^ background. (A-D) Maximum intensity projections of stitched confocal laser scanning z-stack micrographs of *AG-amiRNA*^*i*^ (*OPpro*:*AG-amiRNA/35Spro*:*GR-LhG4*) *FAMApro*:*2xYFP* at (A-B) stage 12 and (C-D) stage 13 gynoecia after (A, C) dexamethasone or (B, D) mock treatments at the times indicated. Scale is 100 μm.
(TIF)

S5 Fig Confocal and epifluorescence imaging of *MUTEpro*:*MUTE-GFP* in L-*er* and *ag-10* backgrounds. (A-F) Images of early stage 12 gynoecial valves of *MUTEpro*:*MUTE-GFP* in (A-C) a wild-type L-*er* background and (D-F) an *ag-10* background using confocal microscopy. Arrowheads indicate the presence of fluorescent foci. (G-O) Images of *MUTEpro*:*MUTE-GFP* (G-L) stage 11 gynoecia in (G-I) a wild-type L-*er* background, (J-L) an *ag-10* background, and (M-O) stage 12 gynoecia in a wild-type L-*er* background. Each panel is a sample from a different plant. Scale is 100 μm.
(TIF)

S6 Fig Sequences bound in *MUTE* first intron by AG-SEP3 from seq-DAP-seq. Sequences mapping to the *MUTE* first intron identified as bound by SEP3 (SEP3_rep1-4), an AG-SEP3 complex (SEP3AG_rep1-3), or an AG-SEP3^Δtet^ complex (SEP3del_AG_rep1-2). CArG_1 and CArG_2 are highlighted in purple boxes.
(TIF)

S7 Fig Stomatal development on gynoecial valves after *AG* knockdown. (A) A stage 13 *ag-10* gynoecia and (B) an enlarged segment of the gynoecial valve depicting the presence of stomatal linage cells with symmetric cell divisions (late-stage GMCs) and mature stomatal cells with a central pore. (C-E) Scanning electron micrographs of gynoecia at anthesis of (C) *AlcRpro*:*AG-amiRNA/35Spro*:*AlcR* 5 d after 6 h EtOH vapor treatment, (D) untreated *AlcRpro*:*AG-amiRNA/35Spro*:*AlcR* and (E) L*-er* 5 d after 6 h EtOH vapor treatment. Scale bars for images of whole gynoecia are 100 μm. Scale bars for magnifications are 20 μm. Purple, blue, and red highlights indicate early, mid, and late-stage stomatal lineage morphology, respectively. (F) Index of early, mid, and late stomatal lineages based on morphology from scanning electron micrographs from stage 13 gynoecial valves of indicated genotypes. Each dot represents an individual sample. NT, no treatment.
(TIF)

S8 Fig RT-qPCRs of *ful-1* and *ag-10* combinations. (A) Levels of *SHP1* and *SHP2* mRNAs as determined by RT-qPCR in L-*er* and *ag-10* stage 10–13 gynoecia. (B-D) Levels of (B) *SPCH*, (C) *MUTE*, and (D) *FAMA* mRNAs as determined by RT-qPCR in L-*er*, *ag-10*, *ful-1*, and *ful-1 ag-10* stage 12–13 gynoecia. Each dot represents the technical mean of an individual independent biological replicate.
(TIF)

S9 Fig Morphology of flowers from mutant *AG* and *SHP* plants. (A-D) Flowers at anthesis of (A) L*-er*, (B) *shp1-1 shp2-1*, (C) *ag-10*, and (D) *ag-10 shp1-1 shp2-1*. Some sepals, petals and stamens have been removed to allow visualization of gynoecium. Scale is 1 mm. (E-F) Levels of (E) *SPCH* and (F) *TMM* mRNAs in L*-er*, *shp1 shp2*, *ag-10*, *ag-10 shp1 shp2* stage 10–13 gynoecia as determined by RT-qPCR. Each dot represents the technical mean of an independent biological replicate.
(TIF)

S10 Fig Gel shift assays of MADS domain transcription factors and the *MUTE* first intron. (A-F) Protein-DNA gel shift assays using combinations of AG, SEP3, SEP3ΔC, SHP1, and SHP2 protein and two replicates of (A) *AtMUTE_i1*, (B) *mCArG_1*, (C) *mCArG_2*, (D) *mCArG_1+2*, (E) *CrMUTE_i1*, and (F) *EsMUTE_i1* probes.
(TIF)

S1 Table Statistical analyses of stomatal indices on the gynoecium/silique valves of L-*er*, *shp1 shp2*, *ag-10*, *ag-10 shp1 shp2*, and *AlcApro*:*AG-amiRNA/35Spro*:*AlcR* or L*-er* based on morphological analysis at different stages of development. (DOCX)

S2 Table Statistical analyses of stomatal indices on the gynoecium/silique valves of L-*er*, *shp1 shp2*, *ag-10*, *ag-10 shp1 shp2*, and *AlcApro*:*AG-amiRNA/35Spro*:*AlcR* or L*-er* based on cell size analysis at different stages of development. (DOCX)

S3 Table Statistical analyses for RT-qPCRs. (DOCX)

S4 Table Statistical analysis of counts of fluorescent foci after repression of *AG* activity. (DOCX)

S5 Table MEME-ChIP input and output. (XLSX)

S6 Table Sequences of probes used for gel shift assays. (DOCX)

S7 Table Output of TBLASTN using AtMUTE amino acid sequence in Phytozome. (XLSX)

S8 Table Statistical analysis of length of L-*er*, *shp1 shp2*, *ag-10*, and *ag-10 shp1 shp2* siliques. (DOCX)

S9 Table Transgenic or mutant plant lines used in this study. (DOCX)

S10 Table Primers used in this study. (DOCX)

S1 Data Underlying numerical data for figures. (XLSX)
