# Supplementary figures and images for: AGAMOUS mediates timing of guard cell formation during gynoecium development
Source: PLoS Genet. 2023 Oct 11;19(10):e1011000. doi: 10.1371/journal.pgen.1011000 (PMC10593234; doi:10.1371/journal.pgen.1011000)

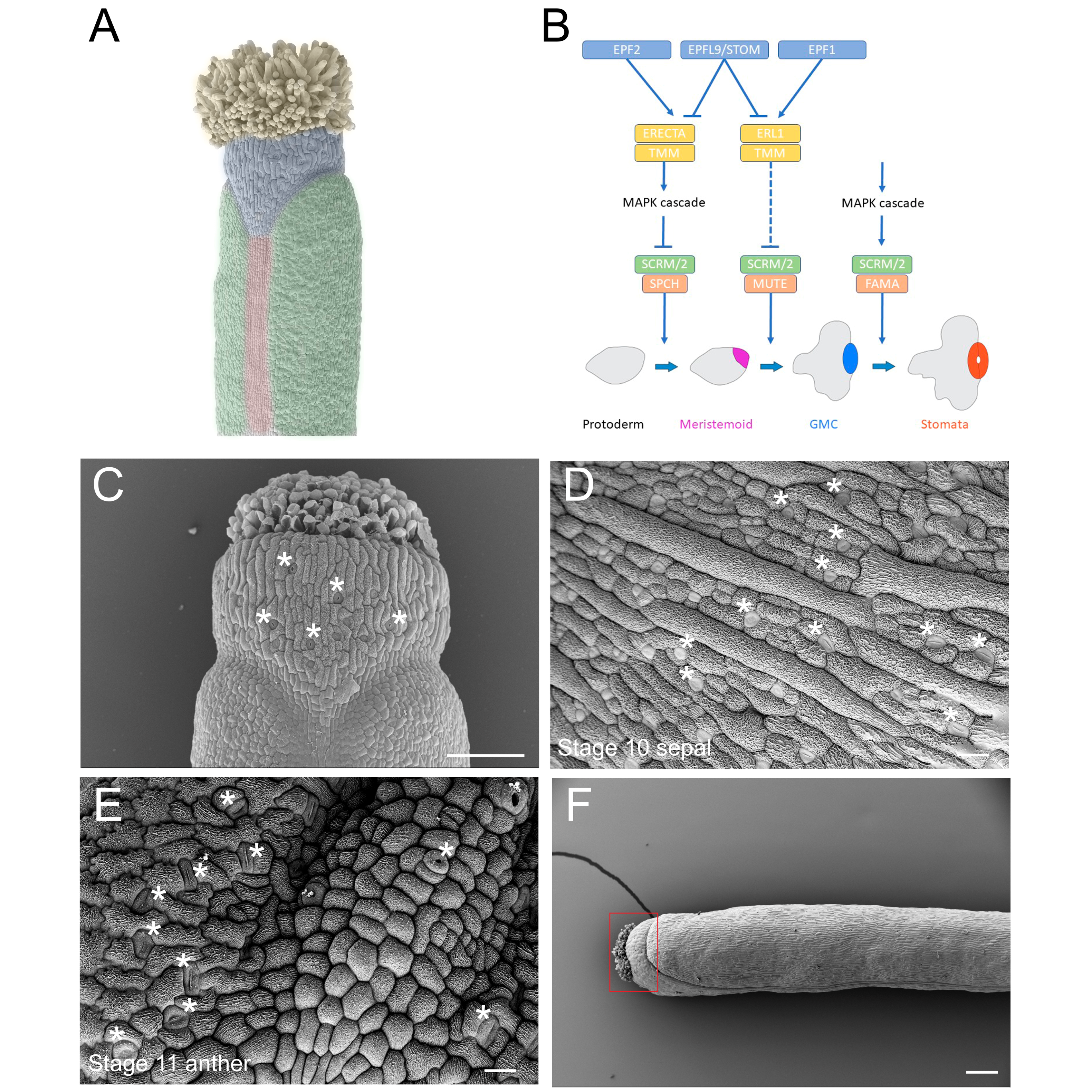

Supplement: S1 Fig — (A) A scanning electron micrograph of a gynoecium at stage 13 (anthesis) with the valves (green), replum (red), style (blue) and stigma (yellow) false colored. (B) Simplified model of stomatal development on leaves. The bHLH transcription factors, SPEECHLESS (SPCH), MUTE, FAMA, SCREAM (SCRM) and SCRM2 coordinate the progression of stomatal development. SPCH is subject to post-translational regulation by a mitogen activated protein kinase (MAPK) cascade, which is activated by receptors, such as TOO MANY MOUTHS (TMM), ERECTA (ER) and ER-LIKEs (ERLs). The secreted peptides, EPIDERMAL PATTERNING FACTOR1 (EPF1) and EPF2 bind to these receptors to activate them at different stages of stomatal development. EPF-LIKE9/STOMAGEN (STOM) competes with EPF1/EPF2 binding to suppress activation of the receptors. The dotted line from the ERL1-TMM complex to the SCRM/2-MUTE complex indicates an unknown mechanism of repression. (C-F) Scanning electron micrographs of (C) a stage 12 gynoecium, (D) a stage 10 abaxial sepal, (E) a stage 11 abaxial anther, and (F) a stage >17 silique. Asterisks indicate presence of stomatal lineage cells. Red box in (F) highlights the size of style tissue in comparison to valve tissue, both of which bear stomata. Scale in (C) 100 μm, (D-E) 20 μm, and (F) 200 μm. (TIF) [file pgen.1011000.s001.tif]

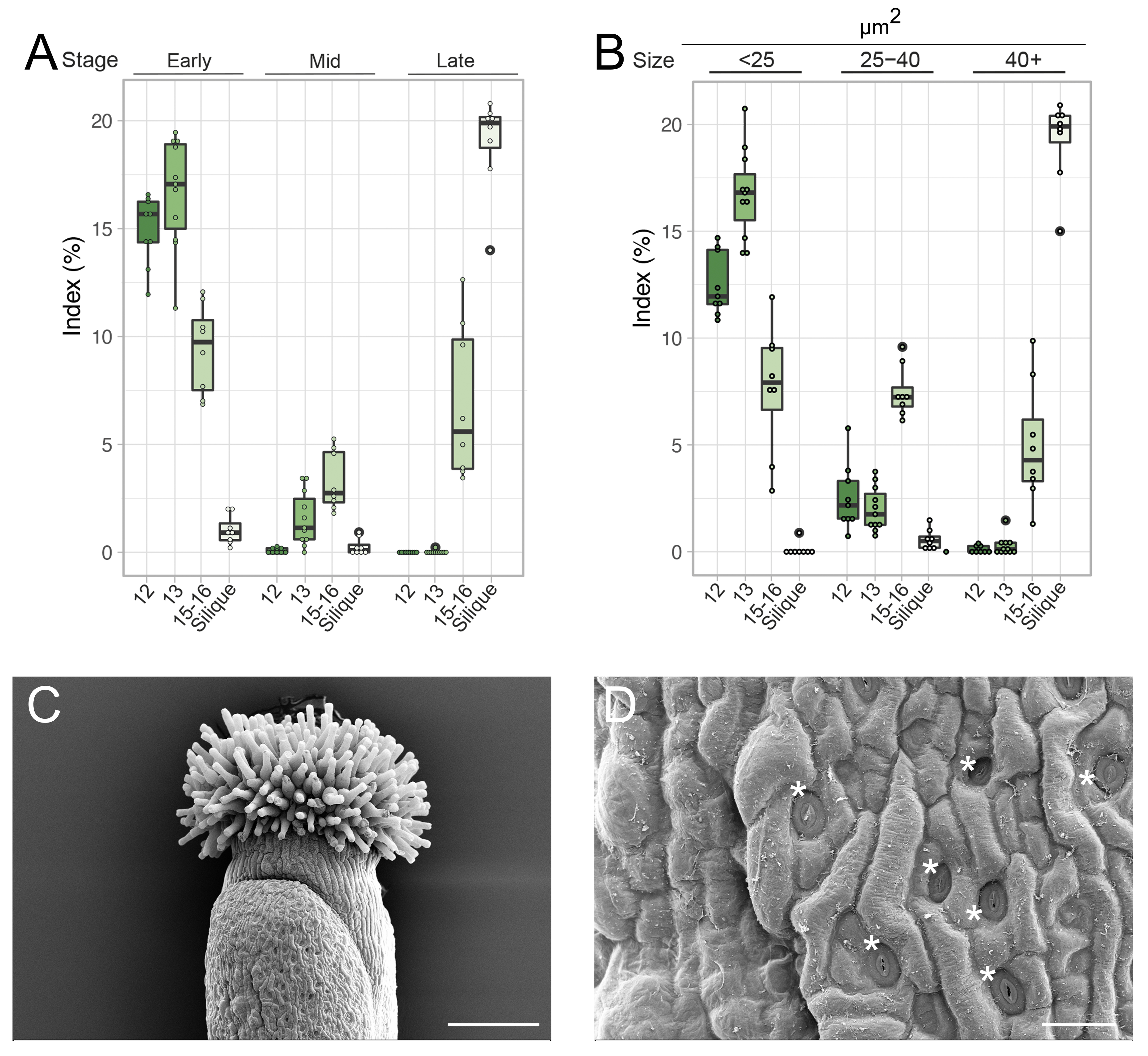

Supplement: S2 Fig — (A-B) The progression of the stomatal lineage on wild-type gynoecia and siliques as determined by (A) morphological assessment and (B) by cell size analysis. (C-D) Scanning electron micrographs of (C) a gynoecium from an emasculated flower 5 days after anthesis, (D) a magnification of the valve in (C). Scale in (C) 200 μm, (D) 20 μm. (TIF) [file pgen.1011000.s002.tif]

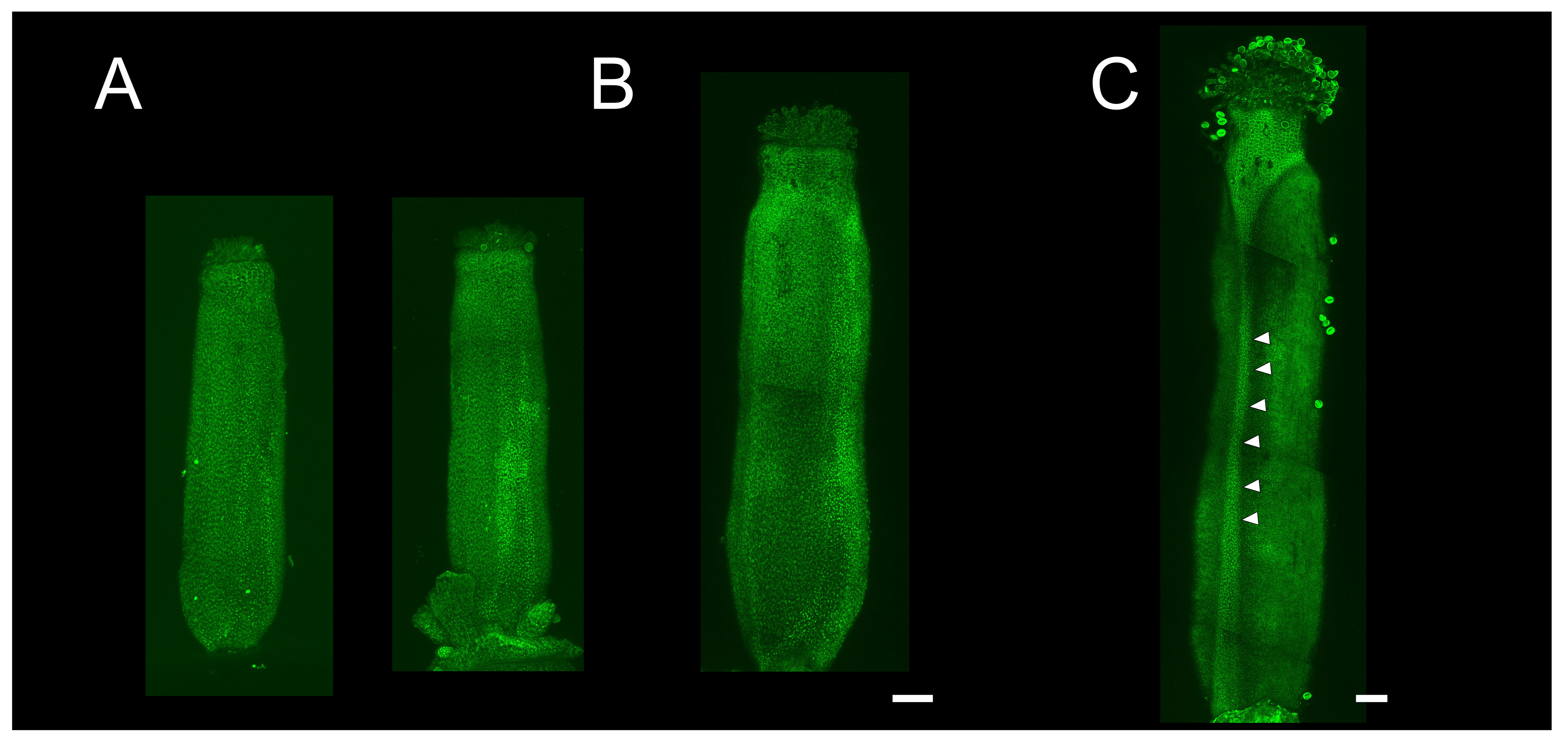

Supplement: S3 Fig — (A-C) Maximum intensity projections of stitched confocal laser scanning z-stack micrographs of (A) early stage 12, (B) late stage 12, and (C) stage 13 gynoecia from AGpro:AG-GFP ag-1 plants. Arrowheads indicate accumulation of AG-GFP in the replum. Scale is 100 μm. (TIF) [file pgen.1011000.s003.tif]

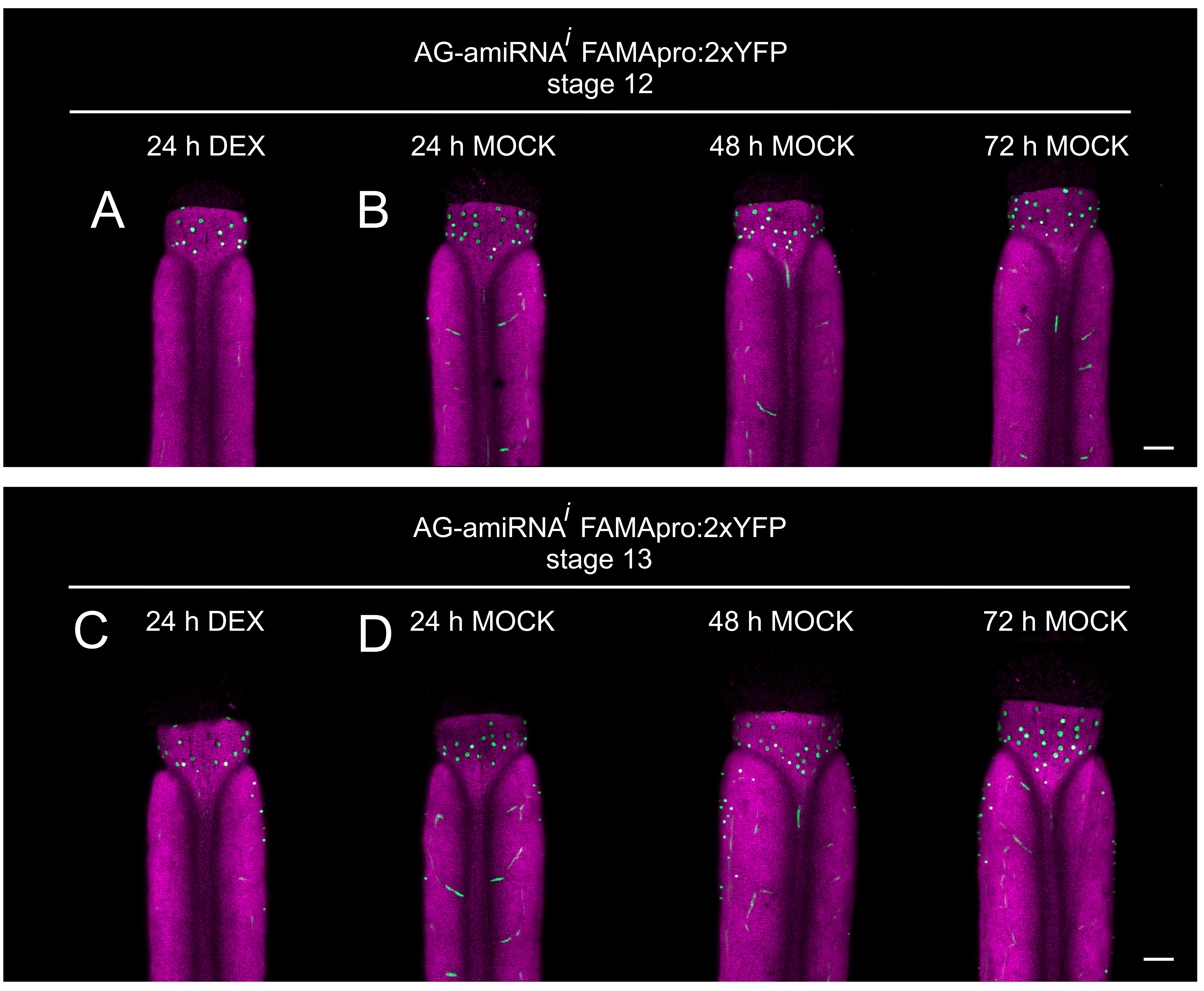

Supplement: S4 Fig — (A-D) Maximum intensity projections of stitched confocal laser scanning z-stack micrographs of AG-amiRNAi (OPpro:AG-amiRNA/35Spro:GR-LhG4) FAMApro:2xYFP at (A-B) stage 12 and (C-D) stage 13 gynoecia after (A, C) dexamethasone or (B, D) mock treatments at the times indicated. Scale is 100 μm. (TIF) [file pgen.1011000.s004.tif]

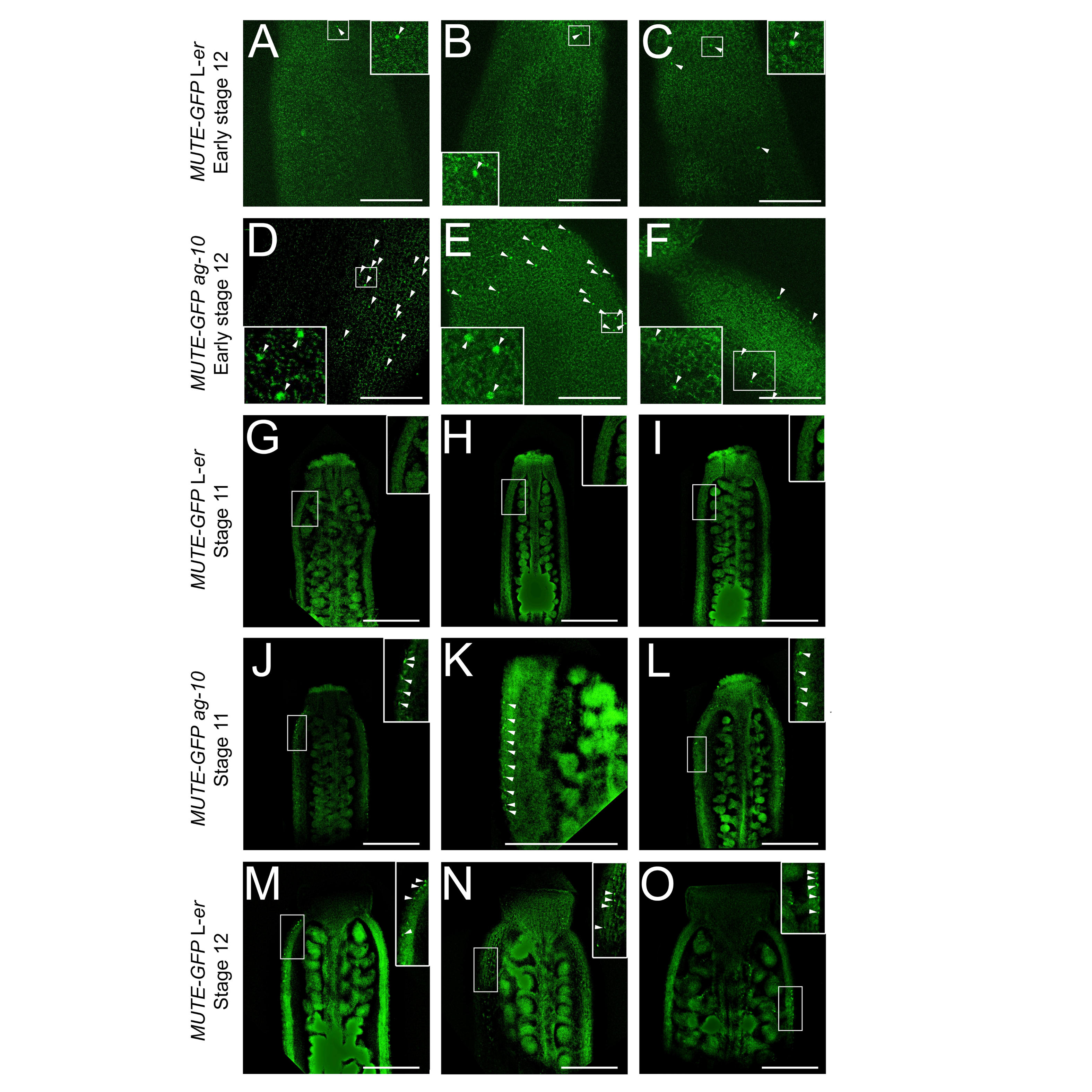

Supplement: S5 Fig — (A-F) Images of early stage 12 gynoecial valves of MUTEpro:MUTE-GFP in (A-C) a wild-type L-er background and (D-F) an ag-10 background using confocal microscopy. Arrowheads indicate the presence of fluorescent foci. (G-O) Images of MUTEpro:MUTE-GFP (G-L) stage 11 gynoecia in (G-I) a wild-type L-er background, (J-L) an ag-10 background, and (M-O) stage 12 gynoecia in a wild-type L-er background. Each panel is a sample from a different plant. Scale is 100 μm. (TIF) [file pgen.1011000.s005.tif]

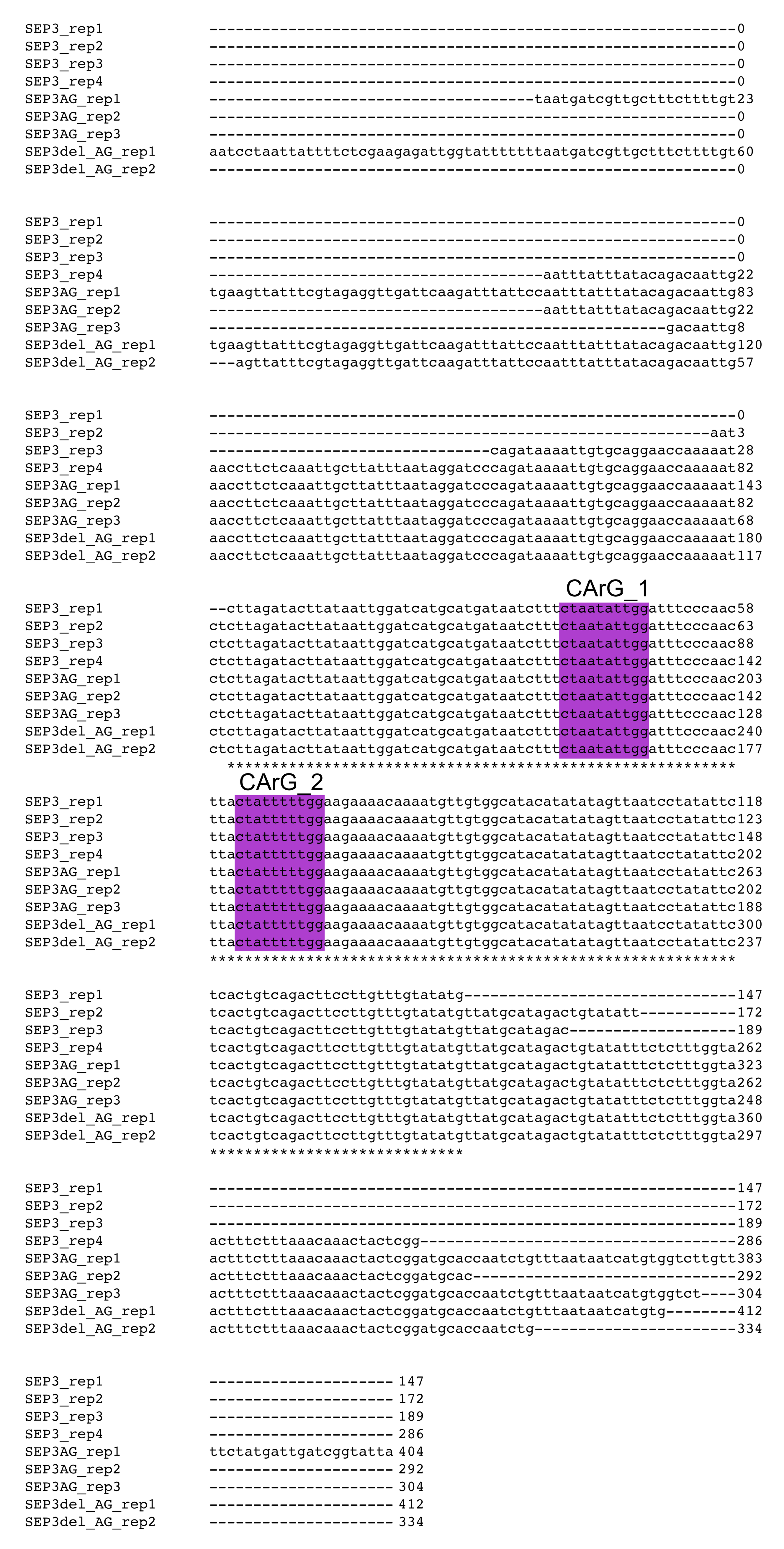

Supplement: S6 Fig — Sequences mapping to the MUTE first intron identified as bound by SEP3 (SEP3_rep1-4), an AG-SEP3 complex (SEP3AG_rep1-3), or an AG-SEP3Δtet complex (SEP3del_AG_rep1-2). CArG_1 and CArG_2 are highlighted in purple boxes. (TIF) [file pgen.1011000.s006.tif]

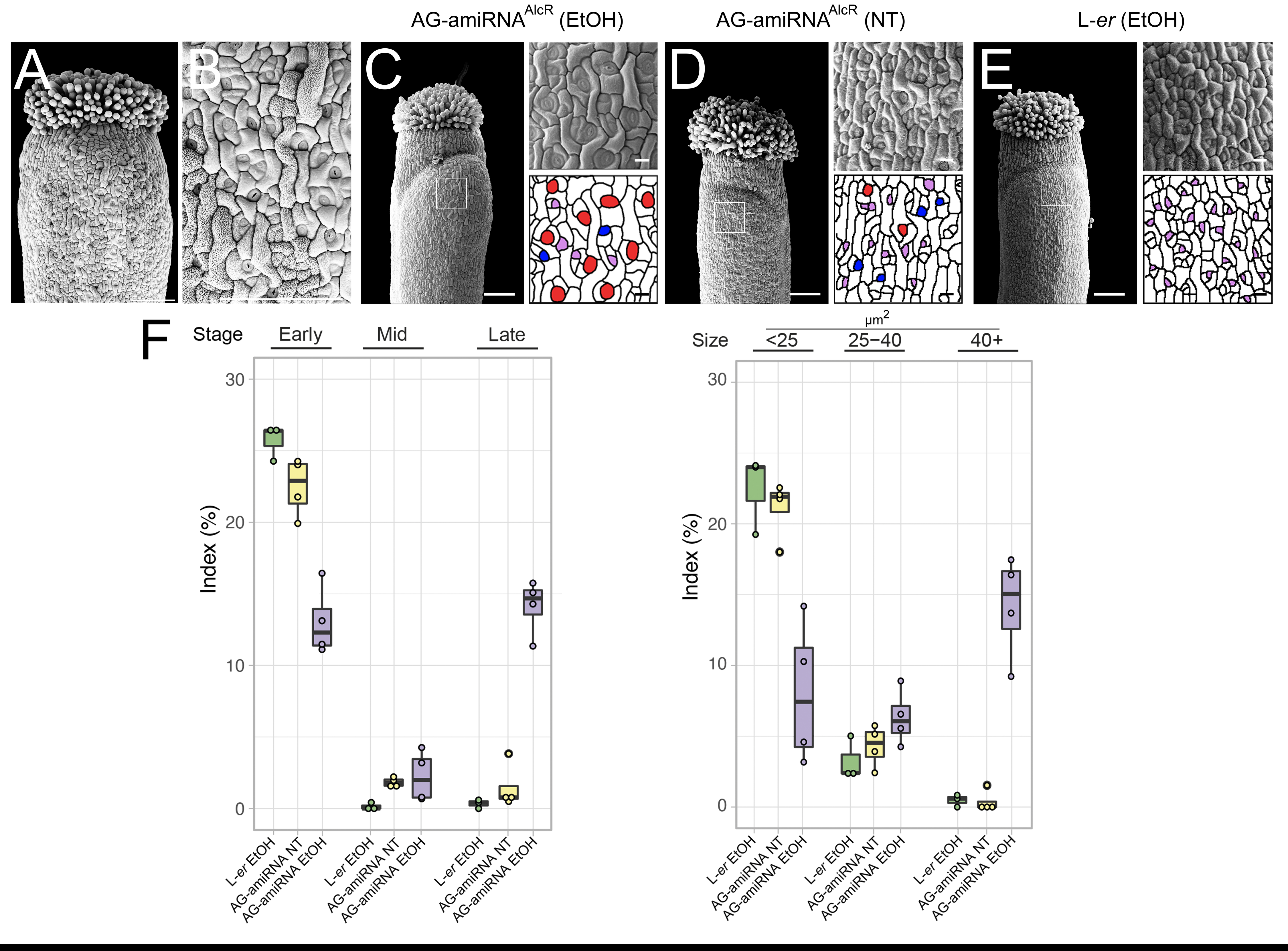

Supplement: S7 Fig — (A) A stage 13 ag-10 gynoecia and (B) an enlarged segment of the gynoecial valve depicting the presence of stomatal linage cells with symmetric cell divisions (late-stage GMCs) and mature stomatal cells with a central pore. (C-E) Scanning electron micrographs of gynoecia at anthesis of (C) AlcRpro:AG-amiRNA/35Spro:AlcR 5 d after 6 h EtOH vapor treatment, (D) untreated AlcRpro:AG-amiRNA/35Spro:AlcR and (E) L-er 5 d after 6 h EtOH vapor treatment. Scale bars for images of whole gynoecia are 100 μm. Scale bars for magnifications are 20 μm. Purple, blue, and red highlights indicate early, mid, and late-stage stomatal lineage morphology, respectively. (F) Index of early, mid, and late stomatal lineages based on morphology from scanning electron micrographs from stage 13 gynoecial valves of indicated genotypes. Each dot represents an individual sample. NT, no treatment. (TIF) [file pgen.1011000.s007.tif]

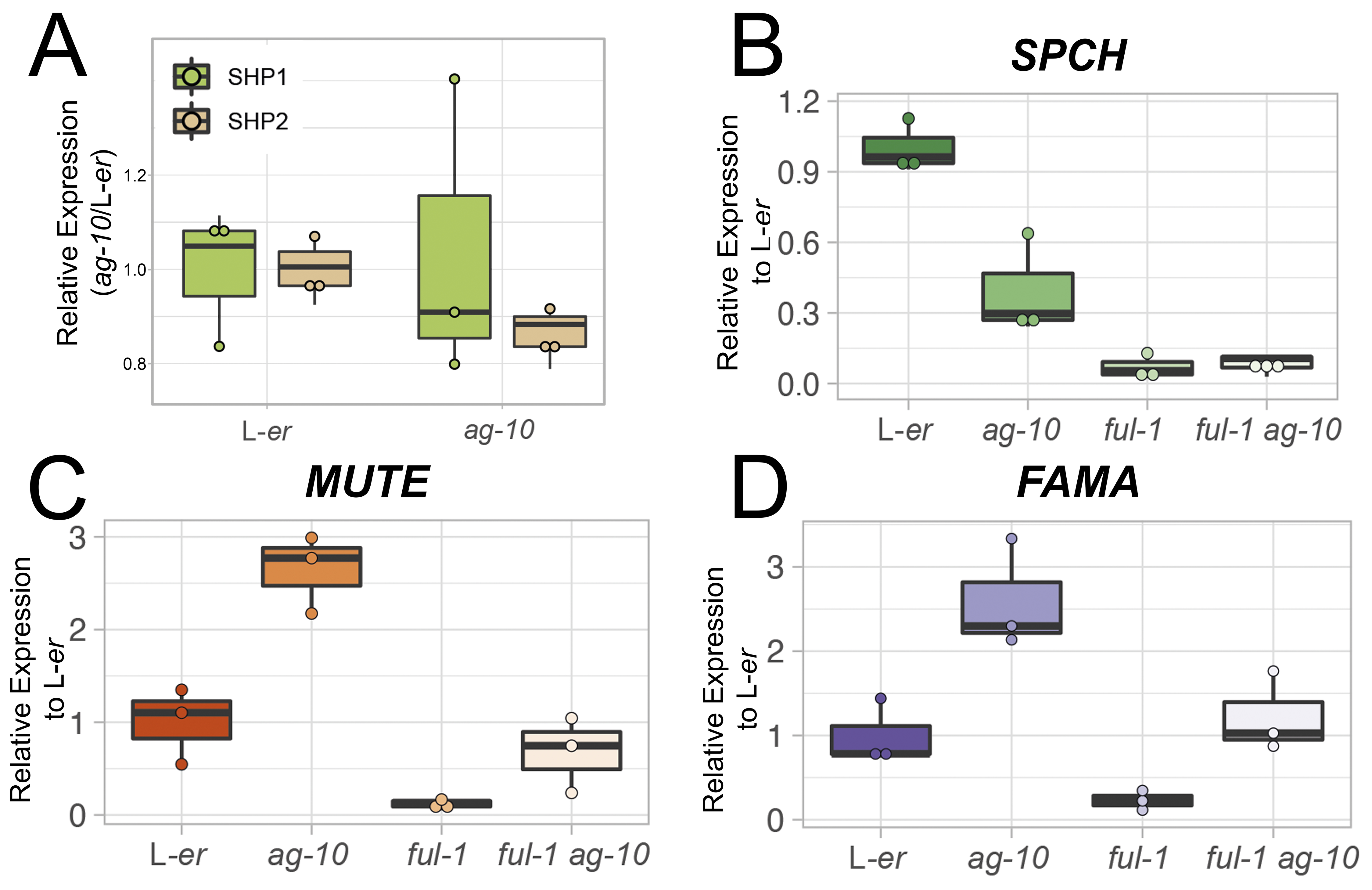

Supplement: S8 Fig — (A) Levels of SHP1 and SHP2 mRNAs as determined by RT-qPCR in L-er and ag-10 stage 10–13 gynoecia. (B-D) Levels of (B) SPCH, (C) MUTE, and (D) FAMA mRNAs as determined by RT-qPCR in L-er, ag-10, ful-1, and ful-1 ag-10 stage 12–13 gynoecia. Each dot represents the technical mean of an individual independent biological replicate. (TIF) [file pgen.1011000.s008.tif]

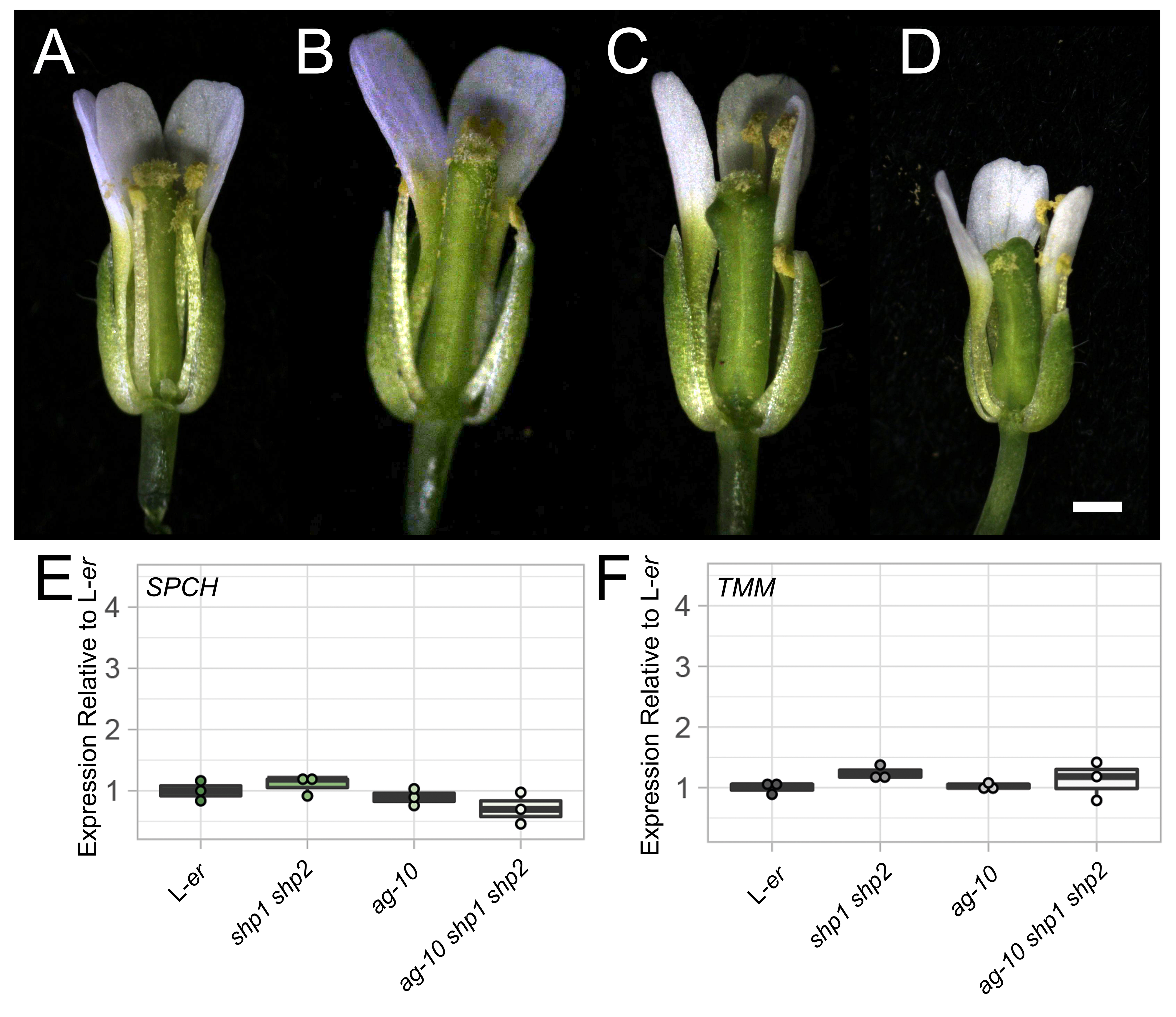

Supplement: S9 Fig — (A-D) Flowers at anthesis of (A) L-er, (B) shp1-1 shp2-1, (C) ag-10, and (D) ag-10 shp1-1 shp2-1. Some sepals, petals and stamens have been removed to allow visualization of gynoecium. Scale is 1 mm. (E-F) Levels of (E) SPCH and (F) TMM mRNAs in L-er, shp1 shp2, ag-10, ag-10 shp1 shp2 stage 10–13 gynoecia as determined by RT-qPCR. Each dot represents the technical mean of an independent biological replicate. (TIF) [file pgen.1011000.s009.tif]

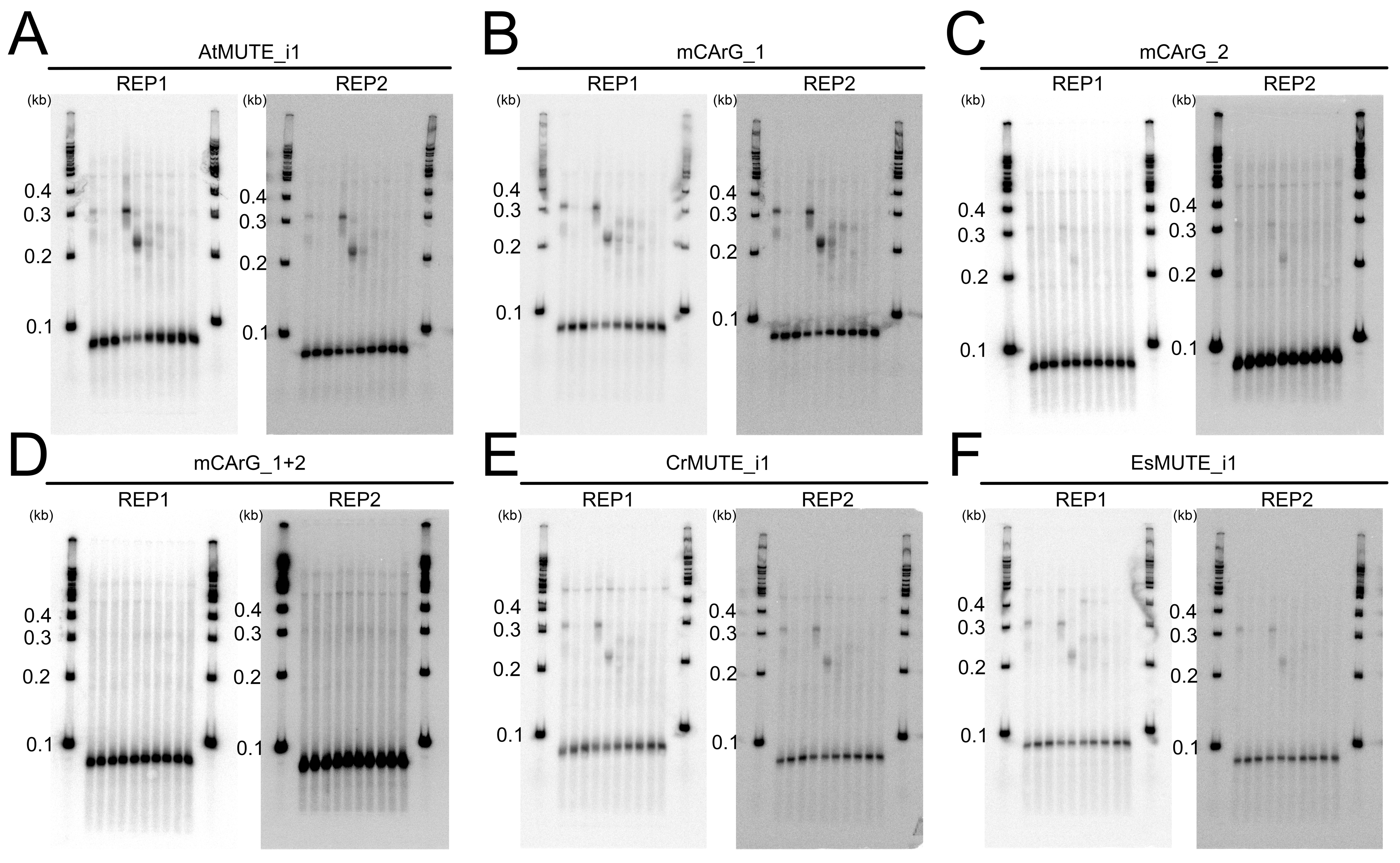

Supplement: S10 Fig — (A-F) Protein-DNA gel shift assays using combinations of AG, SEP3, SEP3ΔC, SHP1, and SHP2 protein and two replicates of (A) AtMUTE_i1, (B) mCArG_1, (C) mCArG_2, (D) mCArG_1+2, (E) CrMUTE_i1, and (F) EsMUTE_i1 probes. (TIF) [file pgen.1011000.s010.tif]
