# Supplementary material for: AGAMOUS mediates timing of guard cell formation during gynoecium development
Source: PLoS Genet. 2023 Oct 11;19(10):e1011000. doi: 10.1371/journal.pgen.1011000 (PMC10593234; doi:10.1371/journal.pgen.1011000)
Supplement: S1 Table — (DOCX) [file pgen.1011000.s011.docx]

| Genotype | Early | Mid | Late | N |
| --- | --- | --- | --- | --- |
|  | Mean ± SD | | |  |
| Stage 12 | | | | |
| L-*er* | 14.94 ± 1.6^*^ | 0.09 ± 0.12^a^ | 0.00 ± 0.00^a^ | 9 |
| *ag-10* | 15.68 ± 1.03^*^ | 0.77 ± 0.28^b^ | 0.34 ± 0.27^a^ | 4 |
| *ag-10 shp1-1 shp2-1* | 14.45 ± 1.78^*^ | 1.15 ± 0.35^b^ | 0.68 ± 0.59^b^ | 4 |
| Stage 13 | | | | |
| L-*er* | 16.66 ± 2.54^a^ | 1.55 ± 1.24^a^ | 0.63 ± 0.98^a^ | 11 |
| *shp1-1 shp2-1* | 14.53 ± 1.18^a^ | 0.29 ± 0.25^b^ | 0.08 ± 0.09^a^ | 8 |
| *ag-10* | 8.48 ± 3.80^b^ | 2.61 ± 1.08^c^ | 8.96 ± 3.98^b^ | 9 |
| *ag-10 shp1-1 shp2-1* | 9.49 ± 1.24^b^ | 3.15 ± 0.67^c^ | 7.07 ± 2.31^b^ | 7 |
| Stage >17 | | | | |
| L-*er* | 1.03 ± 0.67^*^ | 0.26 ± 0.38^*^ | 18.99 ± 2.22^*^ | 8 |
| *shp1-1 shp2-1* | 0.56 ± 0.28^*^ | 2.17 ± 1.66^*^ | 17.53 ± 2.36^*^ | 4 |
| *ag-10* | 0.89 ± 0.75^*^ | 0.15 ± 0.31^*^ | 18.74 ± 2.56^*^ | 12 |
| *ag-10 shp1-1 shp2-1* | 1.43 ± 0.53^*^ | 1.47 ± 1.76* | 18.72 ± 3.07^*^ | 5 |
| Stage 15-16 | | | | |
| L-*er* | 9.41 ± 2.05 | 3.30 ± 1.37 | 6.89 ± 3.57 | 8 |
| Stage 13 | | | | |
| AG-amiRNA*^i^* (EtOH) | 13.04 ± 2.43^a^ | 2.23 ± 1.78^*^ | 14.12 ± 1.94^a^ | 4 |
| AG-amiRNA*^i^* (NT) | 22.49 ± 2.05^b^ | 1.83 ± 0.32^*^ | 1.47 ± 1.58^b^ | 4 |
| L-*er* (EtOH) | 25.71 ± 1.25^b^ | 0.14 ± 0.24^*^ | 0.34 ± 0.30^b^ | 3 |

**Supplemental Table 1. Statistical analyses of stomatal indices on the gynoecium/silique valves of L-*er*, *shp1 shp2*, *ag-10*, *ag-10 shp1 shp2*, and *AlcApro:AG-amiRNA/35Spro:AlcR* or L*-er* based on morphological analysis at different stages of development.** Superscript letters indicate statistical grouping based on pairwise t-tests followed by Benjamini-Hochberg correction for multiple testing (*p* < 0.01). Superscript asterisks indicate that no difference in the means was detected by the one-way ANOVA (*p* > 0.01).
