# Supplementary material for: AGAMOUS mediates timing of guard cell formation during gynoecium development
Source: PLoS Genet. 2023 Oct 11;19(10):e1011000. doi: 10.1371/journal.pgen.1011000 (PMC10593234; doi:10.1371/journal.pgen.1011000)
Supplement: S2 Table — (DOCX) [file pgen.1011000.s012.docx]

| Genotype | <25 μm^2^ | 25-40 μm^2^ | 40+ μm^2^ | N |
| --- | --- | --- | --- | --- |
|  | Mean ± SD | | |  |
| Stage 12 | | | | |
| L-*er* | 12.51 ± 1.46^*^ | 2.54 ± 1.54^*^ | 0.10 ± 0.16^a^ | 9 |
| *ag-10* | 14.75 ± 1.32^*^ | 1.67 ± 1.08^*^ | 0.43 ± 0.26^b^ | 4 |
| *ag-10 shp1-1 shp2-1* | 14.23 ± 2.27^*^ | 1.59 ± 0.45^*^ | 0.47 ± 0.25^b^ | 4 |
| Stage 13 | | | | |
| L-*er* | 16.74 ± 2.08^a^ | 2.00 ± 1.02^a^ | 0.28 ± 0.44^a^ | 11 |
| *shp1-1 shp2-1* | 13.53 ± 1.17^b^ | 1.21 ± 0.38^a^ | 0.15 ± 0.16^a^ | 8 |
| *ag-10* | 10.79 ± 4.18^c^ | 4.70 ± 1.53^b^ | 4.51 ± 2.50^b^ | 9 |
| *ag-10 shp1-1 shp2-1* | 11.55 ± 1.53^bc^ | 4.85 ± 0.89^b^ | 3.29 ± 1.83^b^ | 7 |
| Stage >17 | | | | |
| L-*er* | 0.11 ± 0.31^*^ | 0.58 ± 0.46^*^ | 19.24 ± 1.96^*^ | 8 |
| *shp1-1 shp2-1* | 0.08 ± 0.10^*^ | 0.64 ± 0.36^*^ | 17.58 ± 2.02^*^ | 4 |
| *ag-10* | 0.24 ± 0.51^*^ | 0.65 ± 1.04^*^ | 18.71 ± 2.56^*^ | 12 |
| *ag-10 shp1-1 shp2-1* | 0.21 ± 0.25^*^ | 1.61 ± 1.22^*^ | 16.93 ± 4.05^*^ | 5 |
| Stage 15-16 | | | | |
| L-*er* | 7.66 ± 2.99 | 7.47 ± 1.18 | 4.99 ± 2.85 | 8 |
| Stage 13 | | | | |
| AG-amiRNA*^i^* (EtOH) | 8.06 ± 5.11^a^ | 6.32 ± 1.96^a^ | 14.19 ± 3.67^a^ | 4 |
| AG-amiRNA*^i^* (NT) | 21.10 ± 2.08^b^ | 4.31 ± 1.47^b^ | 0.38 ± 0.77^b^ | 4 |
| L-*er* (EtOH) | 22.45 ± 2.78^b^ | 3.26 ± 1.53^b^ | 0.48 ± 0.43^b^ | 3 |

**Supplemental Table 2. Statistical analyses of stomatal indices on the gynoecium/silique valves of L-*er*, *shp1 shp2*, *ag-10*, *ag-10 shp1 shp2*, and *AlcApro:AG-amiRNA/35Spro:AlcR* or L*-er* based on cell size analysis at different stages of development.** Values reported are the fraction of total cells binned into each cell size interval. Superscript letters indicate statistical grouping based on pairwise t-tests followed by Benjamini-Hochberg correction for multiple testing (*p* < 0.05). Superscript asterisks indicate that no difference in the means was detected by the one-way ANOVA (*p* > 0.05).
