# Supplementary material for: AGAMOUS mediates timing of guard cell formation during gynoecium development
Source: PLoS Genet. 2023 Oct 11;19(10):e1011000. doi: 10.1371/journal.pgen.1011000 (PMC10593234; doi:10.1371/journal.pgen.1011000)
Supplement: S3 Table — (DOCX) [file pgen.1011000.s013.docx]

**Supplemental Table 3. Statistical analyses for RT-qPCRs.**

Experiment 1 – RT-qPCRs L-*er* late stage 11/early stage 12, late stage 12, stage 13, and stage 15-16. Superscript letters indicate statistical grouping based on pairwise t-tests followed by Benjamini-Hochberg correction for multiple testing (*p* < 0.05).

| **Genotype** | **Stage** | **Gene** | **Mean ± SD** | **N** |
| --- | --- | --- | --- | --- |
| L-*er* | Late 11/Early 12 | *AG* | 1.43 ± 0.19^a^ | 4 |
| L-*er* | Late 12 | *AG* | 1.46 ± 0.09^a^ | 4 |
| L-*er* | 13 | *AG* | 1.00 ± 0.08^b^ | 4 |
| L-*er* | 15-16 | *AG* | 0.69 ± 0.09^c^ | 4 |
| L-*er* | Late 11/Early 12 | *SEP3* | 7.17 ± 1.35^a^ | 4 |
| L-*er* | Late 12 | *SEP3* | 8.40 ± 0.18^a^ | 4 |
| L-*er* | 13 | *SEP3* | 6.32 ± 0.28^ab^ | 4 |
| L-*er* | 15-16 | *SEP3* | 7.16 ± 0.53^ab^ | 4 |
| L-*er* | Late 11/Early 12 | *SPCH* | 0.60 ± 0.14^a^ | 4 |
| L-*er* | Late 12 | *SPCH* | 0.78 ± 0.07^b^ | 4 |
| L-*er* | 13 | *SPCH* | 0.57 ± 0.06^a^ | 4 |
| L-*er* | 15-16 | *SPCH* | 0.02 ± 0.01^c^ | 4 |
| L-*er* | Late 11/Early 12 | *MUTE* | 0.02 ± 0.01^ad^ | 4 |
| L-*er* | Late 12 | *MUTE* | 0.06 ± 0.004^be^ | 4 |
| L-*er* | 13 | *MUTE* | 0.20 ± 0.01^c^ | 4 |
| L-*er* | 15-16 | *MUTE* | 0.07 ± 0.03^de^ | 4 |
| L-*er* | Late 11/Early 12 | *FAMA* | 0.03 ± 0.01^a^ | 4 |
| L-*er* | Late 12 | *FAMA* | 0.09 ± 0.01^b^ | 4 |
| L-*er* | 13 | *FAMA* | 0.09 ± 0.01^b^ | 4 |
| L-*er* | 15-16 | *FAMA* | 0.11 ± 0.02^b^ | 4 |

Experiment 2 – RT-qPCRs L-*er* and *ag-10* stage 10-13 gynoecia. Superscript letters indicate statistical grouping based on t-tests (paired, two-tailed, *p* < 0.05).

| **Genotype** | **Gene** | **Mean ± SD** | **N** |
| --- | --- | --- | --- |
| L-*er* | *SPCH* | 0.46 ± 0.12^a^ | 6 |
| *ag-10* | *SPCH* | 0.37 ± 0.14^a^ | 6 |
| L-*er* | *MUTE* | 0.03 ± 0.01^a^ | 6 |
| *ag-10* | *MUTE* | 0.10 ± 0.02^b^ | 6 |
| L-*er* | *FAMA* | 0.07 ± 0.01^a^ | 6 |
| *ag-10* | *FAMA* | 0.13 ± 0.01^b^ | 6 |
| L-*er* | *SCRM* | 2.55 ± 0.21^a^ | 6 |
| *ag-10* | *SCRM* | 2.60 ± 0.40^a^ | 6 |
| L-*er* | *SCRM2* | 1.75 ± 0.36^a^ | 6 |
| *ag-10* | *SCRM2* | 1.99 ± 0.51^a^ | 6 |
| L-*er* | *ERL1* | 1.78 ± 0.21^a^ | 6 |
| *ag-10* | *ERL1* | 1.49 ± 0.10^b^ | 6 |
| L-*er* | *ERL2* | 1.39 ± 0.15^a^ | 6 |
| *ag-10* | *ERL2* | 1.29 ± 0.13^a^ | 6 |
| L-*er* | *TMM* | 1.38 ± 0.17^a^ | 6 |
| *ag-10* | *TMM* | 1.31 ± 0.12^a^ | 6 |
| L-*er* | *EPF1* | 0.02 ± 0.01^a^ | 6 |
| *ag-10* | *EPF1* | 0.07 ± 0.01^b^ | 6 |
| L-*er* | *STOM* | 0.35 ± 0.17^a^ | 6 |
| *ag-10* | *STOM* | 0.57 ± 0.22^b^ | 6 |
| L-*er* | *SHP1* | 2.24 ± 0.33^a^ | 3 |
| *ag-10* | *SHP1* | 2.32 ± 0.72^a^ | 3 |
| L-*er* | *SHP2* | 1.24 ± 0.09^a^ | 3 |
| *ag-10* | *SHP2* | 1.07 ± 0.08^a^ | 3 |

Experiment 3 - RT-qPCRs L-er, *shp1 shp2*, ag*-10*, and *ag-10 shp1 shp2* stage 10-13 gynoecia. Superscript letters indicate statistical grouping based on pairwise t-tests followed by Benjamini-Hochberg correction for multiple testing (*p* < 0.05). Superscript asterisks indicate that no difference in the means was detected by the one-way ANOVA (*p* > 0.05).

| **Genotype** | **Gene** | **Mean ± SD** | **N** |
| --- | --- | --- | --- |
| L-*er* | *SPCH* | 0.54 ± 0.09^*^ | 3 |
| *shp1 shp2* | *SPCH* | 0.60 ± 0.09^*^ | 3 |
| *ag-10* | *SPCH* | 0.49 ± 0.07^*^ | 3 |
| *ag-10 shp1 shp2* | *SPCH* | 0.39 ± 0.14^*^ | 3 |
| L-*er* | *MUTE* | 0.05 ± 0.02^a^ | 6 |
| *shp1 shp2* | *MUTE* | 0.04 ± 0.01^a^ | 6 |
| *ag-10* | *MUTE* | 0.12 ± 0.01^b^ | 6 |
| *ag-10 shp1 shp2* | *MUTE* | 0.16 ± 0.03^c^ | 6 |
| L-*er* | *FAMA* | 0.07 ± 0.01^a^ | 3 |
| *shp1 shp2* | *FAMA* | 0.10 ± 0.01^a^ | 3 |
| *ag-10* | *FAMA* | 0.14 ± 0.002^b^ | 3 |
| *ag-10 shp1 shp2* | *FAMA* | 0.20 ± 0.01^c^ | 3 |
| L-*er* | *SCRM* | 2.51 ± 0.23^*^ | 3 |
| *shp1 shp2* | *SCRM* | 2.62 ± 0.25^*^ | 3 |
| *ag-10* | *SCRM* | 2.49 ± 0.32^*^ | 3 |
| *ag-10 shp1 shp2* | *SCRM* | 1.92 ± 0.48^*^ | 3 |
| L-*er* | *SCRM2* | 1.43 ± 0.05^*^ | 3 |
| *shp1 shp2* | *SCRM2* | 1.64 ± 0.12^*^ | 3 |
| *ag-10* | *SCRM2* | 1.62 ± 0.17^*^ | 3 |
| *ag-10 shp1 shp2* | *SCRM2* | 1.70 ± 0.34^*^ | 3 |
| L-*er* | *ERL1* | 1.85 ± 0.17^a^ | 3 |
| *shp1 shp2* | *ERL1* | 1.87 ± 0.07^a^ | 3 |
| *ag-10* | *ERL1* | 1.56 ± 0.06^a^ | 3 |
| *ag-10 shp1 shp2* | *ERL1* | 0.73 ± 0.13^b^ | 3 |
| L-*er* | *ERL2* | 1.50 ± 0.11^a^ | 3 |
| *shp1 shp2* | *ERL2* | 1.66 ± 0.14^b^ | 3 |
| *ag-10* | *ERL2* | 1.30 ± 0.05^a^ | 3 |
| *ag-10 shp1 shp2* | *ERL2* | 0.82 ± 0.11^c^ | 3 |
| L-*er* | *TMM* | 1.33 ± 0.13^*^ | 3 |
| *shp1 shp2* | *TMM* | 1.65 ± 0.16^*^ | 3 |
| *ag-10* | *TMM* | 1.36 ± 0.08^*^ | 3 |
| *ag-10 shp1 shp2* | *TMM* | 1.50 ± 0.42^*^ | 3 |
| L-*er* | *EPF1* | 0.03 ± 0.01^a^ | 3 |
| *shp1 shp2* | *EPF1* | 0.02 ± 0.003^a^ | 3 |
| *ag-10* | *EPF1* | 0.08 ± 0.01^b^ | 3 |
| *ag-10 shp1 shp2* | *EPF1* | 0.11 ± 0.01^c^ | 3 |
| L-*er* | *STOM* | 0.23 ± 0.002^a^ | 3 |
| *shp1 shp2* | *STOM* | 0.28 ± 0.05^a^ | 3 |
| *ag-10* | *STOM* | 0.40 ± 0.03^a^ | 3 |
| *ag-10 shp1 shp2* | *STOM* | 0.45 ± 0.10^a^ | 3 |

Experiment 4 – RT-qPCRs L-*er* and *ag-10* late stage 11/early stage 12, late stage 12, stage 13, and stage 15-16 gynoecia. Superscript letters indicate statistical grouping based on t-tests (paired, two-tailed, *p* < 0.05).

| **Genotype** | **Stage** | **Gene** | **Mean ± SD** | **N** |
| --- | --- | --- | --- | --- |
| L-*er* | Late 11/Early 12 | *SPCH* | 0.60 ± 0.14^a^ | 4 |
| *ag-10* | Late 11/Early 12 | *SPCH* | 0.71 ± 0.14^b^ | 4 |
| L-*er* | Late 12 | *SPCH* | 0.78 ± 0.07^a^ | 4 |
| *ag-10* | Late 12 | *SPCH* | 0.35 ± 0.04^b^ | 4 |
| L-*er* | 13 | *SPCH* | 0.57 ± 0.06^a^ | 4 |
| *ag-10* | 13 | *SPCH* | 0.24 ± 0.03^b^ | 4 |
| L-*er* | 15-16 | *SPCH* | 0.02 ± 0.01^a^ | 4 |
| *ag-10* | 15-16 | *SPCH* | 0.02 ± 0.002^a^ | 4 |
| L-*er* | Late 11/Early 12 | *MUTE* | 0.02 ± 0.01^a^ | 4 |
| *ag-10* | Late 11/Early 12 | *MUTE* | 0.08 ± 0.01^b^ | 4 |
| L-*er* | Late 12 | *MUTE* | 0.06 ± 0.004^a^ | 4 |
| *ag-10* | Late 12 | *MUTE* | 0.22 ± 0.02^b^ | 4 |
| L-*er* | 13 | *MUTE* | 0.20 ± 0.01^a^ | 4 |
| *ag-10* | 13 | *MUTE* | 0.22 ± 0.01^a^ | 4 |
| L-*er* | 15-16 | *MUTE* | 0.07 ± 0.03^a^ | 4 |
| *ag-10* | 15-16 | *MUTE* | 0.04 ± 0.01^a^ | 4 |
| L-*er* | Late 11/Early 12 | *FAMA* | 0.03 ± 0.01^a^ | 4 |
| *ag-10* | Late 11/Early 12 | *FAMA* | 0.06 ± 0.01^b^ | 4 |
| L-*er* | Late 12 | *FAMA* | 0.09 ± 0.01^a^ | 4 |
| *ag-10* | Late 12 | *FAMA* | 0.14 ± 0.04^b^ | 4 |
| L-*er* | 13 | *FAMA* | 0.09 ± 0.01^a^ | 4 |
| *ag-10* | 13 | *FAMA* | 0.16 ± 0.02^b^ | 4 |
| L-*er* | 15-16 | *FAMA* | 0.11 ± 0.02^a^ | 4 |
| *ag-10* | 15-16 | *FAMA* | 0.07 ± 0.02^a^ | 4 |

Experiment 5 – RT-qPCRs OPpro:AG-amiRNA/35Spro:GR-LhG4 stage 10-13 gynoecia. Superscript letters indicate statistical grouping based on t-tests (paired, two-tailed, *p* < 0.05).

| **Treatment** | **Gene** | **Mean ± SD** | **N** |
| --- | --- | --- | --- |
| MOCK | *SPCH* | 0.47 ± 0.04^a^ | 3 |
| DEX | *SPCH* | 0.33 ± 0.24^a^ | 3 |
| MOCK | *MUTE* | 0.04 ± 0.003^a^ | 3 |
| DEX | *MUTE* | 0.06 ± 0.01^a^ | 3 |
| MOCK | *FAMA* | 0.08 ± 0.01^a^ | 3 |
| DEX | *FAMA* | 0.07 ± 0.03^a^ | 3 |
| MOCK | *SCRM* | 2.39 ± 0.43^a^ | 3 |
| DEX | *SCRM* | 2.04 ± 0.39^a^ | 3 |
| MOCK | *SCRM2* | 1.38 ± 0.57^a^ | 3 |
| DEX | *SCRM2* | 1.65 ± 0.11^a^ | 3 |
| MOCK | *ERL1* | 1.68 ± 0.74^a^ | 3 |
| DEX | *ERL1* | 1.13 ± 0.57^a^ | 3 |
| MOCK | *ERL2* | 1.26 ± 0.57^a^ | 3 |
| DEX | *ERL2* | 1.02 ± 0.33^a^ | 3 |
| MOCK | *TMM* | 1.42 ± 0.16^a^ | 3 |
| DEX | *TMM* | 1.46 ± 0.19^a^ | 3 |
| MOCK | *EPF1* | 0.03 ± 0.01^a^ | 3 |
| DEX | *EPF1* | 0.05 ± 0.01^a^ | 3 |
| MOCK | *STOM* | 0.27 ± 0.10^a^ | 3 |
| DEX | *STOM* | 0.26 ± 0.09^a^ | 3 |

Experiment 6 – RT-qPCRs OPpro:AG-amiRNA/35Spro:GR-LhG4 13 gynoecia (relative to 0 d). Superscript letters indicate statistical grouping in comparison to 0 d, based on pairwise two-tailed t-tests followed by Benjamini-Hochberg correction for multiple testing (*p* < 0.05).

| **Time-point (d)** | **Gene** | **Mean ± SD** | **N** |
| --- | --- | --- | --- |
| 0 | *AG* | 1.84 ± 0.004^a^ | 2 |
| 1 | *AG* | 0.43 ± 0.04^b^ | 3 |
| 2 | *AG* | 0.34 ± 0.01^b^ | 3 |
| 3 | *AG* | 0.28 ± 0.03^b^ | 3 |
| 4 | *AG* | 0.45 ± 0.08^b^ | 3 |
| 5 | *AG* | 0.46 ± 0.09^b^ | 3 |
| 6 | *AG* | 0.86 ± 0.11^b^ | 3 |
| 7 | *AG* | 0.88 ± 0.18^b^ | 3 |
| 0 | *SPCH* | 0.57 ± 0.06^a^ | 2 |
| 1 | *SPCH* | 0.55 ± 0.10^a^ | 3 |
| 2 | *SPCH* | 0.35 ± 0.10^b^ | 3 |
| 3 | *SPCH* | 0.24 ± 0.08^b^ | 3 |
| 4 | *SPCH* | 0.24 ± 0.04^b^ | 3 |
| 5 | *SPCH* | 0.20 ± 0.05^b^ | 3 |
| 6 | *SPCH* | 0.24 ± 0.004^b^ | 3 |
| 7 | *SPCH* | 0.19 ± 0.01^b^ | 3 |
| 0 | *MUTE* | 0.13 ± 0.06^a^ | 2 |
| 1 | *MUTE* | 0.15 ± 0.03^a^ | 3 |
| 2 | *MUTE* | 0.23 ± 0.03^b^ | 3 |
| 3 | *MUTE* | 0.23 ± 0.02^b^ | 3 |
| 4 | *MUTE* | 0.30 ± 0.02^b^ | 3 |
| 5 | *MUTE* | 0.28 ± 0.03^b^ | 3 |
| 6 | *MUTE* | 0.27 ± 0.03^b^ | 3 |
| 7 | *MUTE* | 0.24 ± 0.03^b^ | 3 |
| 0 | *FAMA* | 0.12 ± 0.05^a^ | 2 |
| 1 | *FAMA* | 0.12 ± 0.02^a^ | 3 |
| 2 | *FAMA* | 0.24 ± 0.08^b^ | 3 |
| 3 | *FAMA* | 0.25 ± 0.05^b^ | 3 |
| 4 | *FAMA* | 0.46 ± 0.03^b^ | 3 |
| 5 | *FAMA* | 0.45 ± 0.02^b^ | 3 |
| 6 | *FAMA* | 0.42 ± 0.01^b^ | 3 |
| 7 | *FAMA* | 0.44 ± 0.06^b^ | 3 |

Experiment 7 – RT-qPCR L-*er*, *ag-10*, *ful-1*, *ag-10 ful-1* stage 12-13 gynoecia. Superscript letters indicate statistical grouping based on pairwise t-tests followed by Benjamini-Hochberg correction for multiple testing (*p* < 0.05). Superscript asterisks indicate that no difference in the means was detected by the one-way ANOVA (*p* > 0.05).

| **Genotype** | **Gene** | **Mean ± SD** | **N** |
| --- | --- | --- | --- |
| L-*er* | *SPCH* | 0.19 ± 0.02^a^ | 3 |
| *ful-1* | *SPCH* | 0.01 ± 0.01^b^ | 3 |
| *ag-10* | *SPCH* | 0.07 ± 0.04^b^ | 3 |
| *ful-1 ag-10* | *SPCH* | 0.02 ± 0.01^b^ | 3 |
| L-*er* | *MUTE* | 0.04 ± 0.02^a^ | 3 |
| *ful-1* | *MUTE* | 0.004 ± 0.002^a^ | 3 |
| *ag-10* | *MUTE* | 0.10 ± 0.02^bc^ | 3 |
| *ful-1 ag-10* | *MUTE* | 0.02 ± 0.01^ac^ | 3 |
| L-*er* | *FAMA* | 0.04 ± 0.01^ab^ | 3 |
| *ful-1* | *FAMA* | 0.01 ± 0.004^b^ | 3 |
| *ag-10* | *FAMA* | 0.10 ± 0.02^ac^ | 3 |
| *ful-1 ag-10* | *FAMA* | 0.05 ± 0.02^b^ | 3 |
