# Supplementary material for: AGAMOUS mediates timing of guard cell formation during gynoecium development
Source: PLoS Genet. 2023 Oct 11;19(10):e1011000. doi: 10.1371/journal.pgen.1011000 (PMC10593234; doi:10.1371/journal.pgen.1011000)
Supplement: S4 Table — (DOCX) [file pgen.1011000.s014.docx]

| **Sample** | **Mean ± SD** | **N** |
| --- | --- | --- |
| *OPpro:AG-amiRNA/35Spro:GR-LhG4* Late-Stage 12 | | |
| 24 h MOCK | 3.00 ± 0.00^*^ | 1 |
| 24 h DEX | 0.00 ± 0.00^*^ | 1 |
| 48 h MOCK | 4.50 ± 0.71^a^ | 2 |
| 48 h DEX | 19.33 ± 3.21^b^ | 3 |
| 72 h MOCK | 0.50 ± 0.71^a^ | 2 |
| 72 h DEX | 83.67 ± 34.44^b^ | 3 |
| Combined MOCK | 2.50 ± 2.38^a^ | 4 |
| Combined DEX | 51.50 ± 41.48^b^ | 6 |
| *OPpro:AG-amiRNA/35Spro:GR-LhG4* Early-Stage 13 | | |
| 24 h MOCK | 3.00 ± 0.00^*^ | 1 |
| 24 h DEX | 5.00 ± 0.00^*^ | 1 |
| 48 h MOCK | 11.00 ± 8.49^a^ | 2 |
| 48 h DEX | 83.33 ± 22.14^b^ | 3 |
| 72 h MOCK | 34 ± 25.46^a^ | 2 |
| 72 h DEX | 261.33 ± 81.33^b^ | 3 |
| Combined MOCK | 22.50 ± 20.40^a^ | 4 |
| Combined DEX | 172.33 ± 111.12^b^ | 6 |
| *OPpro:AG-amiRNA/35Spro:GR-LhG4* Combined Stages | | |
| Combined MOCK | 12.50 ± 17.18^a^ | 8 |
| Combined DEX | 111.92 ± 101.86^b^ | 12 |
| Late Stage 12 | | |
| L-*er* | 3.67 ± 4.62^a^ | 3 |
| *ag-10* | 83.67 ± 42.44^a^ | 3 |
| Stage 13 | | |
| L-*er* | 7.00 ± 6.24^a^ | 3 |
| *ag-10* | 182.67 ± 32.25^b^ | 3 |

**Supplemental Table 4. Statistical analysis of counts of fluorescent foci after repression of AG activity.** Superscript letters indicate statistical grouping based on two-tailed paired or unpaired t-tests (*p* < 0.05). Asterisks indicate samples that were not statistically analysed.
