# Supplementary material for: AGAMOUS mediates timing of guard cell formation during gynoecium development
Source: PLoS Genet. 2023 Oct 11;19(10):e1011000. doi: 10.1371/journal.pgen.1011000 (PMC10593234; doi:10.1371/journal.pgen.1011000)
Supplement: S6 Table — (DOCX) [file pgen.1011000.s016.docx]

**Supplemental Table 6. Sequences of probes used for gel shift assays.** Mutations introduced in CArG motifs are underlined.

Motifs with similarity to CArG motifs are in bold. Sequences are orientated 5`-3`.

AtMUTE_i1 CTCGAGgatcatgcatgataatcttt**ctaatattgg**atttcccaactta**ctatttttgg**aagaaaacaaaatgttgtggTCTAGA

mCArG_1 CTCGAGgatcatgcatgataatctttgacagttttaatttcccaactta**ctatttttgg**aagaaaacaaaatgttgtggTCTAGA

mCArG_2 CTCGAGgatcatgcatgataatcttt**ctaatattgg**atttcccaacttatttatgctgtaagaaaacaaaatgttgtggTCTAGA

mCArG_1+2 CTCGAGgatcatgcatgataatctttgacagttttaatttcccaacttatttatgctgtaagaaaacaaaatgttgtggTCTAGA

EsMUTE_i1 CTCGAGgatcatgcatggtaatcttt**ctaatatcgg**atagtactccaaactta**ctaatcttgg**aaaaatatgttgtgccatctTCTAGA

CrMUTE_i1 CTCGAGgatcatgcatggttaccct**ttaatactgg**atatccccgactta**ctaatttcgg**aaaaatatgttatgatcatgTCTAGA
