# Supplementary material for: AGAMOUS mediates timing of guard cell formation during gynoecium development
Source: PLoS Genet. 2023 Oct 11;19(10):e1011000. doi: 10.1371/journal.pgen.1011000 (PMC10593234; doi:10.1371/journal.pgen.1011000)
Supplement: S8 Table — (DOCX) [file pgen.1011000.s018.docx]

**Supplemental Table 8. Statistical analysis of length of L-*er*, *shp1 shp2*, *ag-10*, and *ag-10 shp1 shp2* siliques.** Superscript letters indicate statistical grouping based on pairwise t-tests followed by Benjamini-Hochberg correction for multiple testing (*p* < 0.05).

| **Genotype** | **Mean ± SD (mm)** | **N** |
| --- | --- | --- |
| L-*er* | 13.30 ± 1.16^a^ | 25 |
| *shp1 shp2* | 12.19 ± 1.27^b^ | 25 |
| *ag-10* | 9.37 ± 1.54^c^ | 25 |
| *ag-10 shp1 shp2* | 4.57 ± 0.77^d^ | 25 |
