# Supplementary material for: AGAMOUS mediates timing of guard cell formation during gynoecium development
Source: PLoS Genet. 2023 Oct 11;19(10):e1011000. doi: 10.1371/journal.pgen.1011000 (PMC10593234; doi:10.1371/journal.pgen.1011000)
Supplement: S9 Table — (DOCX) [file pgen.1011000.s019.docx]

**Supplemental Table 9. Transgenic or mutant plant lines used in this study.**

| **Genotype** | **Reference** |
| --- | --- |
| *shp1-1 shp2-1* | (Liljegren *et al.*, 2000) |
| *ag-10* | (Liu *et al.*, 2011) |
| *ag-10 shp1-1 shp2-1* | (This study) |
| *ful-1* | (Gu *et al.*, 1998) |
| *ag-10 ful-1* | (This study) |
| *SPCHpro:SPCH-YFP spch-3* | (Davies and Bergmann, 2014) |
| *MUTEpro:MUTE-GFP mute-1* | (Pillitteri *et al.*, 2007) |
| *FAMApro:2xYFP* | (Lee *et al.*, 2019) |
| *FAMApro:NLS-2xYFP* | (Adrian *et al.*, 2015) |
| *FAMApro:2xYFP* (L-*er* backcross) | (This study) |
| *FAMApro:2xYFP ag-10* | (This study) |
| *AGpro:AG-GFP ag-1* | (Ó’Maoiléidigh *et al.*, 2013) |
| *OPpro:AG-amiRNA/35Spro:GR-LhG4* | (Ó’Maoiléidigh *et al.*, 2013) |
| *AlcApro:AG-amiRNA/35Spro:AlcR* | (Ó’Maoiléidigh *et al.*, 2013) |
| *MUTEpro:MUTE-GFP* (L-*er* backcross) | (This study) |
| *MUTEpro:MUTE-GFP ag-10* | (This study) |
