# Supplementary material for: AGAMOUS mediates timing of guard cell formation during gynoecium development
Source: PLoS Genet. 2023 Oct 11;19(10):e1011000. doi: 10.1371/journal.pgen.1011000 (PMC10593234; doi:10.1371/journal.pgen.1011000)
Supplement: S10 Table — (DOCX) [file pgen.1011000.s020.docx]

**Supplemental Table 10. Primers used in this study.**

| **Name** | **Sequence (5` to 3`)** | **Purpose** |
| --- | --- | --- |
| spch-3_F | ACAACGAACGAGGAAGAGAGAG | Genotyping |
| spch-3_R | ACTCTCGGGCTTAGGACTTCG | Genotyping |
| SAIL_LB1 | GCCTTTTCAGAAATGGATAAATAGCCTTGCTTCC | Genotyping |
| MUTE_in2-CDS_F | catgtgcagATAGGATTGGAGTGTC | Genotyping |
| sGFP_F | AGGAGCGCACCATCTTCTTC | Genotyping |
| sGFP_R | TCCAGCAGGACCATGTGATC | Genotyping |
| YFP_F | AGTTCATCTGCACCACCGGCAAG | Genotyping |
| YFP_R | ACTCCAGCAGGACCATGTGATCG | Genotyping |
| FUL | CTCATGAGCTTTCTTGAGC | Genotyping |
| ful-1_F | TGTATTCACGTCACATACCG | Genotyping |
| ful-1_R | CTTGTAACGCGCTTTCCC | Genotyping |
| *ag-10* | CAATGTCTCCCAAAGAGCCCAGGAACTT | Genotyping |
| *ag-10* | GCAACAAGGCATATAGATTTAATTTG | Genotyping |
| DM_shp1-1_F | GTCTACTGATGAGTTGTCACTAGG | Genotyping |
| DM_shp1-1_R | GTGACGGAAGGAGGGTTGACG | Genotyping |
| DM_shp2-1_F | GAGGATAGAGAACACTACGAATCGTC | Genotyping |
| DM_shp2-1_R | CAGGTCAAGTCAATAGATTCCCTAC | Genotyping |
| DM_shp1-1_T-DNA | GATGCACTCGAAATCAGCCAATTTTAGAC | Genotyping |
| SPCH_qRT_F | TTAGCCCGCGCATCAACCAC | RT-qPCR |
| SPCH_qRT_R | GGAGGCTGTGGGATGAGTGGTAG | RT-qPCR |
| MUTE_qRT_F | GGGCAGCTCGTAAAGATCATCTCTG | RT-qPCR |
| MUTE_qRT_R | TGAACTTCAAGAGTTAGCTCCTCCAAGC | RT-qPCR |
| FAMA_qRT_F | CGAACCAAGCACAACCGCTC | RT-qPCR |
| FAMA_qRT_R | AGCAGCTTCACCTCCACGTC | RT-qPCR |
| SCRM_qRT_F | GGGTCTGTTGCTCGCTACCATG | RT-qPCR |
| SCRM_qRT_R | TATCTCTTGTCCTTCTTGGCATTGCTC | RT-qPCR |
| SCRM2_qRT_F | CGCTGAGCAATGTCAAGAAGACCATG | RT-qPCR |
| SCRM2_qRT_R | TCCACACAGATGCTTCCTTCTGC | RT-qPCR |
| ERL1_qRT_F | CCTTGTCTCCTACTGGCAACCTTC | RT-qPCR |
| ERL1_qRT_R | CGCTATCTTCAACCTTGTCTCCCAATC | RT-qPCR |
| ERL2_qRT_F | AGTAATGGAAGCTGTTGATGCAGAGG | RT-qPCR |
| ERL2_qRT_R | CCTAGAGACCTCCTGCATGGTGG | RT-qPCR |
| STOM_qRT_F | CCCTTCTCCCACAAGTACATCTCCTG | RT-qPCR |
| STOM_qRT_R | AAGCACTGTTGATAGGGTCATTTCCTTC | RT-qPCR |
| TMM_qRT_F | TGGAGGATGAGGAGAAAGCTGAGG | RT-qPCR |
| TMM_qRT_R | GGAGCTGGTCTCGACGTTTCTG | RT-qPCR |
| AG_qRT_F | ACCAGATTCTTCGTGCAAAGATAGCTG | RT-qPCR |
| AG_qRT_R | AAGCTGCTCGTAGTTAGATCCTCCTG | RT-qPCR |
| SEP3_qRT_F | GGGTATCAGATGCCACTCCAGC | RT-qPCR |
| SEP3_qRT_R | GCTCCCATTCCATCTTGTTGCC | RT-qPCR |
| SHP1_qRT_F | AGAATGAGCTGTTAGTGGCAGAGATAGAG | RT-qPCR |
| SHP1_qRT_R | ATTCCTGCTGGTCCGGATTCAATC | RT-qPCR |
| SHP2_qRT_F | GAAGCACGAGATGTTAGTTGCAGAGATTG | RT-qPCR |
| SHP2_qRT_R | CTCGATTCTTGTTGCTGTAGACCTGTTC | RT-qPCR |
| REF1_At1g13320_qRT_F | AAGCGGTTGTGGAGAACATGATACG | RT-qPCR |
| REF1_At1g13320_qRT_R | TGGAGAGCTTGATTTGCGAAATACCG | RT-qPCR |
